# New Insight into the Mechanism of Neurochemical Imbalance in Multiple Sclerosis: Abnormal Transportation of Brain Extracellular Space

**DOI:** 10.14336/AD.2024.1444

**Published:** 2026-02-11

**Authors:** Yumeng Cheng, Jiao Liu, Feng Tian, Hanbo Tan, Tianyu Wang, Jiabin Lu, Zeqing Tang, Xinlei Ma, Jingge Lian, Shaoyi Su, Yu Fu, Bin Liu, Yuliang Li, Wanyi Fu, Meng Xu, Hongbin Han

**Affiliations:** ^1^Department of Radiology, Peking University Third Hospital, Beijing, China.; ^2^Institute of Medical Technology, Peking University Health Science Center, Beijing, China.; ^3^Peking University Third Hospital, Beijing Key Laboratory of Magnetic Resonance Imaging Devices and Technology, Beijing, China.; ^4^Center of Medical and Health Analysis, Peking University Health Science Center, Beijing, China.; ^5^Department of Laboratory Animal Science, Peking University Health Science Center, Beijing, China.; ^6^Department of Interventional Medicine and Minimally Invasive Oncology, The Second Hospital of Shandong University, Jinan, Shandong, China.; ^7^NMPA key Laboratory for Evaluation of Medical Imaging Equipment and Technique, Beijing, China.

**Keywords:** extracellular space, interstitial fluid, multiple sclerosis, volume transmission, neurochemical imbalance

## Abstract

Neurochemical imbalance is a contributing factor to neurological symptoms in multiple sclerosis (MS). The matured myelin sheath is crucial for substance transportation within the extracellular space (ECS) and for maintaining local homeostasis. Therefore, we hypothesize that disturbed ECS transportation following demyelinating lesions might lead to neurochemical imbalance in MS. In the current study, a lysophosphatidylcholine-induced unilateral MS model was used to investigate spatial neurochemical alterations. The results demonstrated that 168 substances were altered around the demyelination site in the ipsilateral hemisphere, compared to the contralateral hemisphere, with significant enrichment in the purine and arginine-proline metabolic pathways. Notably, dopamine was unexpectedly detected in the demyelinated region and the adjacent thalamus. Tracer-based MRI further revealed that the tracer injected into the striatum abnormally refluxed to the thalamus, with the area of reflux consistent with the altered dopamine distribution. The interstitial fluid drained extensively but was confined to the unilateral hemisphere, which may explain the observed widespread changes in other neuroactive substances. Importantly, after the restoration of ECS integrity, both interstitial fluid drainage and neurochemical imbalance, including dopamine, were normalized, supporting the potential link between ECS dysfunction and neurochemical imbalance. These observations highlight the crucial role of ECS transport in maintaining neurochemical homeostasis in the brain, providing new insights into the mechanisms that may underline the neuropsychiatric symptoms of MS.

## INTRODUCTION

Multiple sclerosis (MS) is a prevalent demyelinating disease of the central nervous system, often accompanied by neuropsychiatric symptoms such as fatigue, depression, cognitive impairment, and schizophrenia [1, 2]. Neurochemical imbalance is thought to play a critical role in the pathophysiology of MS [3, 4]. However, its mechanism remains unclear. Neuroactive substances are released into the brain extracellular space (ECS), yet limited research has focused on the specific environments in which these substances exert their effects. Previous studies indicate that ECS homeostasis is critical for brain functions [5, 6]. The potential contribution of ECS destabilization to neurochemical imbalance has yet to be explored.

Mature and intact myelin serves as a structural and partitioning barrier within the brain ECS [5]. In this study, we induced focal demyelination in the internal capsule of rats [7] to disrupt normal molecular transportation within the ECS. Spatial metabolomics profiling revealed an imbalance in various neuroactive substances following demyelination, with an observed enrichment in purine and arginine-proline metabolic pathways. Dopamine signals were detected not only in the striatum but also at the demyelinated site and in the adjacent thalamus. Concurrently, tracer-based MRI revealed that interstitial fluid (ISF) in striatum abnormally refluxed to the adjacent thalamus, while the area of abnormal reflux coincided with the regions exhibiting these dopamine signals. The unilateral spread of deep brain ISF was extensive within the ipsilateral hemisphere, although it did not extend to the contralateral side. Given the metabolite differences observed between the two hemispheres in the unilateral model, we propose a new hypothesis: ISF drainage may drive and amplify changes in neurochemical imbalance in MS. By elucidating the relationship between ECS integrity, ISF dynamics, and neurochemical balance, this study provides new insights into the mechanisms underlying neurochemical imbalance in MS.

## MATERIALS AND METHODS

### Animals

This study was approved by the Ethics Committee of Peking University Health Science Center (LA2020356). All procedures were conducted in accordance with the Guidelines for the Care and Use of Laboratory Animals at Peking University Health Science Center. Male Sprague-Dawley rats (weighing 250-280 g) were used in this study and randomly assigned to either the control or lysophosphatidylcholine (LPC) group. All animals were obtained from the Department of Laboratory Animal Science at Peking University Health Science Center and housed under barrier conditions. They had ad libitum access to clean-grade rodent chow. The animals were maintained on a 12-hour light/dark cycle at a temperature of 20-25°C and humidity of 55%-65%.

### Experimental Design and Grouping

**Figure 1.**
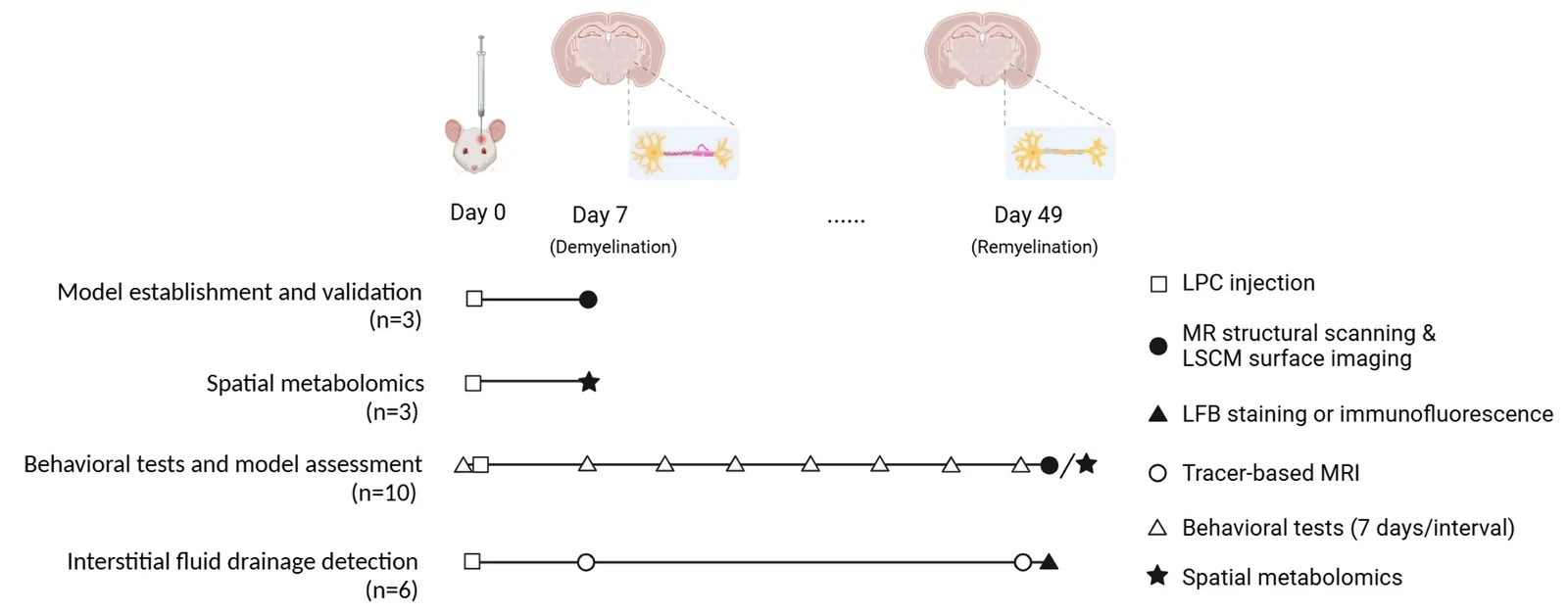
**Experimental design and schematic figure of the study**. The behavioral testing group (n=10) underwent baseline behavioral assessments on day 0, followed by behavioral paradigm testing every 7 days, with tests conducted in order from low-stress to high-stress paradigms.

The experimental design and workflow are outlined in Figure 1. This study followed the “Reduction” principle of the 3R guidelines (Replacement, Reduction, Refinement) for animal research. Different groups of rats were named according to the injected substance and the time elapsed post-injection. “LPC-D7” represented rats injected with LPC at Day 7, and “Phosphate Buffered Saline (PBS)-D7” group served as its control group.

### Establishment of Demyelinated Rat Model

Rats were anesthetized, and their scalps were shaved and disinfected. An incision was made to expose the cranial sutures. For the LPC group, rats received a stereotaxic injection of 1% LPC (L4129, Sigma Aldrich, dissolved in PBS: HY-K3005, MCE, USA) using a 10μl microinjection syringe (Hamilton 701 N 10µl Syringe 26S/51/3, 80383, Hamilton, Switzerland). The injection was administrated at a rate of 0.1μl/min and a total volume of 1μl at the coordinates AP: -1.5mm, ML: +3mm, DV: -6.5mm. After the injection, the incision was sutured and disinfected. The control group received an equivalent volume of PBS injected at the same coordinates.

### MRI Structural Imaging

Animals were anesthetized with a combination anesthetic of 3ml/kg. Then, they were positioned prone on a soft pad for MRI. We utilized an MRI system with 4 cm surface coil. The scan was performed using T2 Turbo Spin Echo sequence, with acquisition parameters: TR of 2700ms, TE of 83ms, six averages, 192 phase encoding steps, echo train length of 12, flip angle of 120°, acquisition time of 4 minutes 17 seconds, acquisition matrix of 192×192, slice thickness of 1mm, and FOV of 38mm×38mm. Tracer was stereotactically injected into the striatum. The tracers used include 1μl 10mmol/L Gd-DTPA (Magnevist; Bayer Schering Pharma AG, Berlin, Germany) and 1μl 10mmol/L calcein (C0875, Sigma-Aldrich, dissolved in PBS), injected at coordinates AP: +1mm, ML: +3mm, DV: -6mm). The T2 TSE scan was acquired before tracer injection and repeated one hour after. The subsequent images were analyzed u sing the NUMARIS post-processing software (Siemens, Germany). Additionally, tracer distribution in the striatum was evaluated using T1-weighted (T1WI) MRI with the following parameters: TR of 516ms, TE of 13ms, two averages, 160 phase encoding steps, echo train length of 1, flip angle of 90°, acquisition matrix of 192×160, slice thickness of 1mm, and FOV: 38mm×31.7mm.

### Laser Scanning Confocal Microscopy (LSCM) Scanning

Two hours after the calcein injection, the animals were perfused with 4% Paraformaldehyde Fix Solution (SL1830, Coolaber, China) and their brains were post-fixed overnight. The brains were then sliced at a 15° oblique sagittal angle using a 1mm coronal rat brain mold to obtain 2mm thick slices surrounding the lesion site. These slices were affixed to the bottom of a confocal dish with tape for stabilization.

Myelin imaging was performed using an SP8 DIVE microscope (Leica, Germany) equipped with a water immersion objective lens (Leica APO 25×, numerical aperture 0.95) and a 30/70 partially reflective mirror. The imaging utilized excitation light sources at 488, 561, and 633 nm to capture the reflection signals across three channels. These included the 498-550 nm for calcein signal (green), and 559-564 nm (red) and 631-636 nm (purple) for other structures, each within a 5 nm bandwidth. All channels were scanned using the xyz scanning mode.

### LFB-Eosin Staining

Brain tissue slices were dehydrated using a sucrose gradient, embedded in Tissue-Tek® O.C.T. Compound (Sakura, Japan), and flash-frozen in liquid nitrogen. The frozen sections were then baked overnight in an oven at 37° and subsequently washed in phosphate-buffered saline (PBS, HY-K3005, MCE, USA) to remove any OCT residue. The slices were then incubated for 16 hours in Luxol Fast Blue (LFB) solution (G3245, Beijing Solarbio Science & Technology Co., Ltd., China). Once differentiable gray-white matter contrast was achieved, the process was stopped with water. These sections were counterstained with eosin diluted in PBS. followed by dehydration in 95% and 100% ethanol subsequently. They were then cleared in a xylene substitute and mounted with neutral resin. Finaly, the slices were imaged using the Pannoramic MIDI II digital slide scanner (3DHISTECH, Hungary).

### Behavioral tests

Behavioral tests were conducted every 7 days, starting the day before lesion induction to its recovery.

Cylinder Test: The cylinder test was used to assess forelimb asymmetry (n=10). Baseline data were collected before the lesson induction on day 0. Rats were placed in a transparent plastic cylinder with 20cm in diameter and 40cm in height. They were allowed to explore freely while being video-recorded from above for 3 minutes. The number of upward touches made by the rat using the ipsilateral paw, contralateral paw, or both paws was recorded. An asymmetry index was calculated as the ratio of ipsilateral paw touches to contralateral paw touches.

Corner Turn Test: The corner turn test assessed turning asymmetry in rats (n=10). Rats were positioned in a 30° angled apparatus, where whisker stimulation induced them to step back and turn. The direction of each turn, whether left or right, was recorded. Each rat was tested 10 times per session, with sufficient rest intervals. The percentage of turns to the ipsilateral or contralateral side was then calculated.

Rotarod Test: This test assessed overall motor function (n=10), using a rotating rod (ZS-RDM-DS, Beijing Zhongshidichuang Science and Technology Development Co., Ltd.). Before formal testing, rats were trained for four days. The initial speed of the rod was set at 10 rpm, increasing by 5 rpm every 5 seconds up to a maximum speed of 50 rpm. Each rat was tested 5 times with a 15-minute interval between trials. The average time each rat remained before falling off time was recorded, and the mean and standard deviation were calculated.

### Spatial Metabolomics

Spatial metabolomics were examined using air-flow-assisted desorption electrospray ionization (AFADESI) mass spectrometry imaging [8]. To simultaneously visualize the distribution of substances in brain, fresh brain tissue was sectioned at the same oblique sagittal angle in optical imaging using a vibratome (VT1200, Leica). The sections were embedded in Cryo-Gel (39475237, Leica) and flash-frozen in liquid nitrogen. Bilateral brain tissue was collected from animals with unilateral lesions, using the contralateral side as an internal control (n=3). Frozen sections were mounted on Superfrost Plus Slides (Epredia) and vacuum-dried for 1 hour. The mass spectrometry parameters are provided in Supplementary Table 1. After data acquisition, the horizontal and vertical resolution of the imaging was approximately 50μm and 100μm, respectively. The raw files were converted to .cdf format using Xcalibur 3.0 (Thermo Fisher Scientific, USA), and MassImager Image reconstruction, background subtraction, ROI selection, and data extraction was performed using Pro software (developed by the Institute of Materia Medica, Chinese Academy of Medical Sciences, and Comwin Beijing Technology Co., Ltd.). For striatum, internal capsule, thalamus, average mass spectra were obtained by selecting ROIs, and the two-dimensional matrix data of these brain microregion were saved as .txt files. Peak alignment and isotope ion removal were performed using MarkerView 1.2.1 (AB Sciex, USA). Principal component analysis (PCA) was used to process the multidimensional spatial metabolomics dataset, providing an overall description. Orthogonal Partial Least Squares-Discriminant Analysis (OPLS-DA) was performed using SIMCA 14.1 software (Umetrics, Umea, Sweden). Metabolites with Variable Importance in Projection (VIP) values greater than 1 and p-values less than 0.05 in independent sample t-tests were considered differential metabolites, and the metabolites were preliminarily annotated based on their exact mass-to-charge ratio (Δppm <5) using the HMDB 5.0 database. The preliminary annotations were further screened and filtered using an experienced in-house library. Based on the resulting potential differential metabolites, we performed pathway analysis of differential metabolic pathways in the striatum, internal capsule, and thalamus between two groups using the MetaboAnalyst 6.0 Pathway Analysis module (www.metaboanalyst.ca/MetaboAnalyst/. The HMDB IDs of the differential metabolites were matched with compounds in the HMDB, PubChem, and KEGG databases. The Rattus norvegicus (rat) pathway library was selected, using hypergeometric testing for enrichment analysis and relative betweenness centrality for topology analysis.

### Tracer-based MRI

This study builds on tracer-based MRI technique which previously described in reference [9] (n=6). Animals were anesthetized and placed in a 3.0T MRI scanner (Magnetum, Trio, Siemens). Each scan was performed using an 8-channel wrist coil and a 3D MP-RAGE sequence [10]. The acquisition parameters for the 3D MP-RAGE sequence were as follows: TR: 1500ms, TE: 3.7ms, number of averages: 2, phase encoding steps: 96, echo train length: 1, flip angle: 9°, acquisition time: 4 min 48 s, acquisition matrix: 512×96, slice thickness and gap: 0.5mm, FOV: 50mm×267mm. Stereotactic injection of 2μl of 10mmol/L Gd-DTPA was performed at coordinates AP: +1mm, ML: +3mm, DV: -6mm, with an injection rate of 0.2μl/min. After the injection, the needle was left in place for 10 minutes and then slowly withdrawn over 5 minutes. The animal was immediately removed from the stereotactic frame, and the 3D MP-RAGE sequence was repeated. Additional 3D MP-RAGE scans were conducted at 15 min, 30 min, 1h, 1.5h, 2h, 2.5h, 3h, 3.5h, and 4h post-injection to collect a series of images. These images were analyzed using NanoDetect Analyze System (V2.1) to calculate ECS parameters as detailed in reference [11].

### SPECT/CT imaging

2% isoflurane anesthesia was used throughout the experiment (n=3). After anesthesia of male SD rats, the fur was shaved, and the anterior fontanelle was exposed and marked. The rats were fixed on a stereotaxic apparatus, and the cranial surface coordinates of the caudate nucleus were located with the anterior fontanelle as the reference point (anterior: 1.0 mm, right: 3.0 mm, depth: 6.0 mm). The target site was marked, and a hole was drilled in the skull. Using a microinjection syringe under protection by lead glass, ^99m^Tc-DTPA was aspirated for intracranial injection. The injection rate was controlled at 0.2 μl/min for 10 minutes, injecting a total of 2 μl of ^99m^Tc-DTPA. After the injection, the needle was left in place for 5 minutes, and then slowly withdrawn over 10 minutes to prevent backflow along the needle tract. SPECT/CT (NanoSPECT/CT, Mediso) was used to detect the tracer for at least 6 hours.

### Statistical Analysis

Statistical analysis and graphing were performed using GraphPad Prism 9. Prior to conducting statistical tests, data were subjected to normality and homogeneity of variance tests, confirming that all datasets followed a normal distribution with equal variance. Behavioral results were analyzed using two-way ANOVA, followed by Tukey’s test for multiple comparisons. For comparisons between two groups, independent sample t-tests or paired t-tests were used as appropriate. For comparisons among three groups, one-way ANOVA was conducted, with Tukey’s test for multiple comparisons. Data are presented as mean ± standard deviation, and a *P*<0.05 was considered statistically significant.

**Figure 2.**
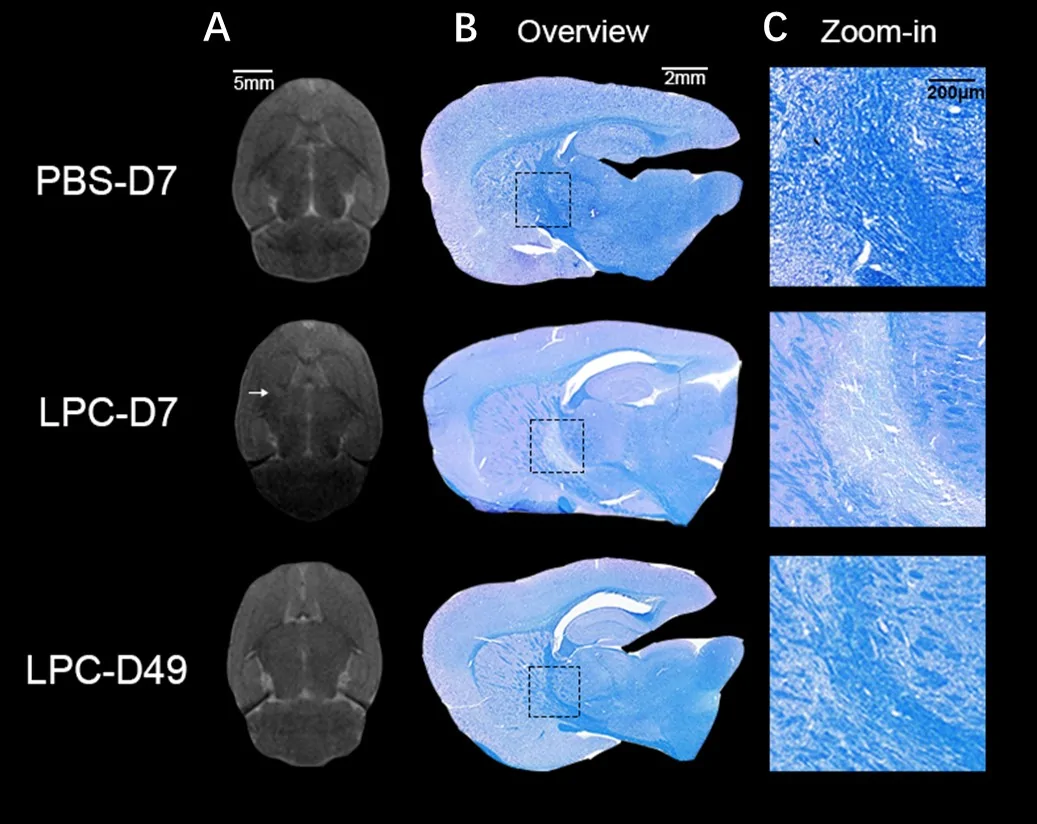
***In vivo* and histological observations of demyelination and remyelination time points in the model**. (**A**) T2 weighted MRI structural scan results. Arrow indicates higher signal intensity compared to the normal internal capsule. (**B**) LFB-eosin myelin staining of frozen sections near the lesion site shows lighter staining at the internal capsule in the LPC-D7 group, while LPC-D49 returned normal. (**C**) Zoom-ins are all magnified images from the dashed box.

## RESULTS

### Unilateral MS rat model demyelinated on day 7 and remyelinated on day 49

LPC was unilaterally injected into the forelimb region of the internal capsule in rats. Previous studies have shown that demyelination peaks at 7 days after LPC injection in mice [7]. We observed similar localized demyelination on day 7 in our rat model. MRI T2 TSE scans revealed elevated signals at the lesion site in the LPC-D7 group compared to the PBS-D7 control group, indicating changes in tissue composition (Fig. 2A). LFB staining of frozen brain sections revealed that while the internal capsule showed normal blue staining in the PBS-D7 group, the LPC-D7 group exhibited significantly lighter staining around the lesion site, indicating myelin loss (Fig. 2C). Interestingly, the lighter staining was not circular but extended along the trajectory of the internal capsule, suggesting the route of LPC metabolism after injection.

**Figure 3.**
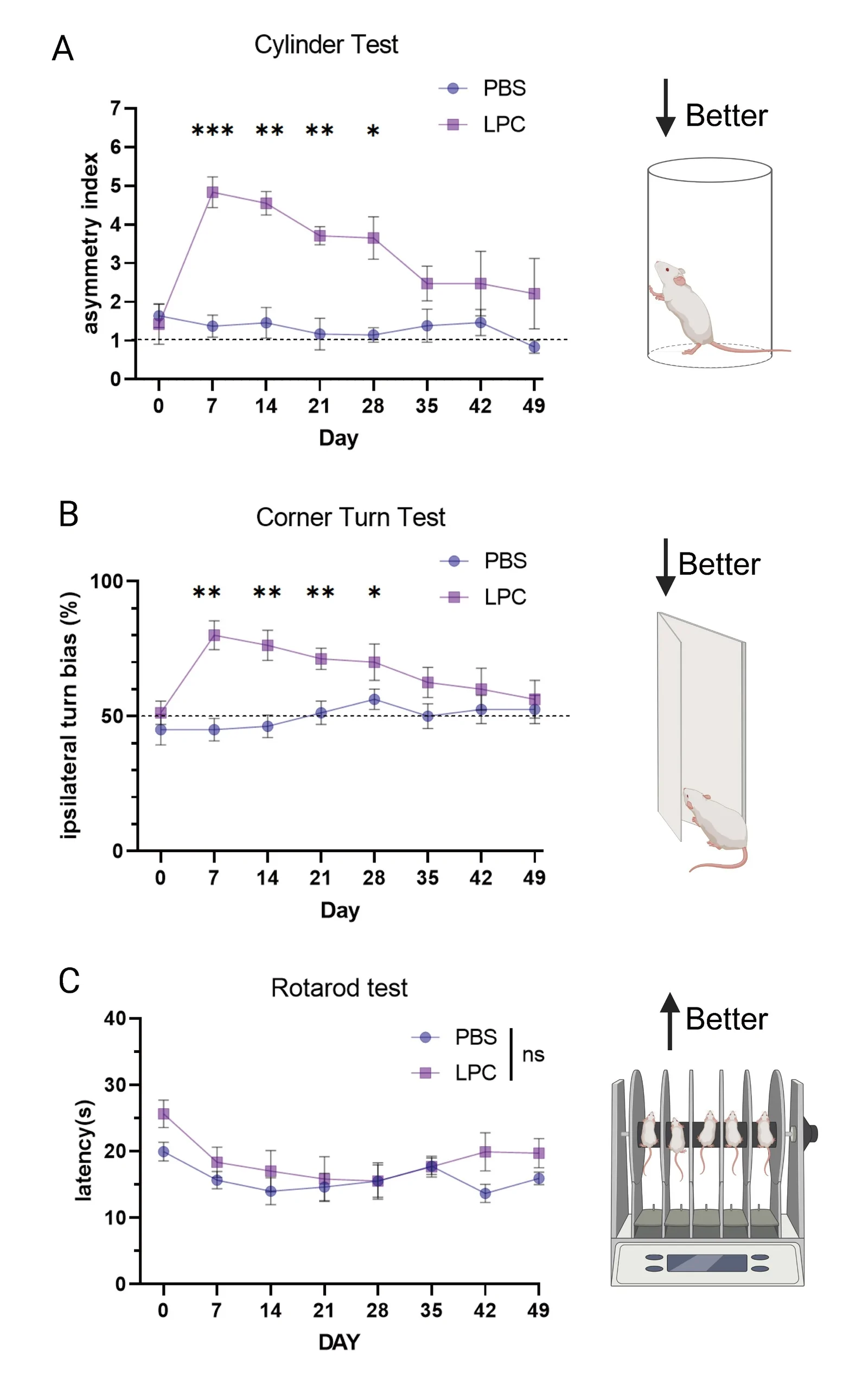
**Cylinder, corner turn, and rotarod tests indicated demyelination followed by remyelination in our model**. (**A**) Asymmetry index in the cylinder test over time for PBS and LPC groups post-modeling (n=10). (**B**) Percentage of ipsilateral turns in the corner turn test over time for PBS and LPC groups post-modeling (n=10). (**C**) Fall latency in the rotarod test over time for PBS and LPC groups post-modeling (n=10). Data analyzed with two-way ANOVA, followed by Tukey’s test for multiple comparisons. Data are presented as mean ± SEM, with dashed lines indicating normal levels. ***P<0.001; **P<0.01; *P<0.05.

The time point of complete remyelination in rats is unclear. We conducted various behavioral tests to gain insights into the development of our model. The internal capsule is a pathway for peripheral sensory conduction and plays a role in regulating limb movement [12]. After damage to the left internal capsule, due to the brain's contralateral control, the motor function on the right side of the body was impaired. In the cylinder test [13], PBS-D7 rats used both forelimbs equally to climb, with an asymmetry index of approximately 1. However, the LPC-D7 group displayed significant forelimb asymmetry, with the average asymmetry index rising to 4.833 (4.833±0.983 vs. 1.373±0.703, *P*=0.0003; two-way ANOVA; n=6). During the following process, the asymmetry index difference between the two groups decreased, indicating gradual recovery of the internal capsule in the LPC group. By day 35, there was no significant difference in asymmetry index between the two groups, indicating forelimb usage of the rats had gradually returned to symmetry (Fig. 3A). Similarly, in the corner turn test [14], rats turn left and right equally when both whiskers are stimulated under normal conditions. However, following demyelination in the LPC group, the proportion of turns to the unaffected side increased drastically. As the remyelination process occurred, ipsilateral turn bias gradually returned to the normal 50%, indicating recovery of internal capsule function (Fig. 3B). No differences were observed between groups at any time point in the rotarod test (adapted from another study [15], Fig. 3C), suggesting that the model used in this study was relatively mild and did not significantly impact overall motor function.

### Spatial metabolomics revealed neurochemical imbalance

We utilized spatial metabolomics (Air Flow-Assisted Desorption Electrospray Ionization Mass Spectrometry Imaging, AFADESI-MSI [16, 17]) to assess brain metabolites differences between two cerebral hemispheres in the LPC-D7 group, as the contralateral side serve as an internal control in our unilateral injection model [18]. We selected the ROIs in the striatum, internal capsule and thalamus, and performed unsupervised PCA analysis on the metabolic profiles in both positive and negative ion modes. The score plots indicated a natural grouping trend of metabolites in the three regions (striatum as an example in Supplementary Fig. 1), suggesting potential differences in metabolites between two sides. Subsequently, OPLS-DA was used to identify differential metabolites, revealing 88, 82, and 38 unique annotated metabolites in the striatum, internal capsule, and thalamus, respectively (Supplementary Tables 2-4).

**Figure 4.**
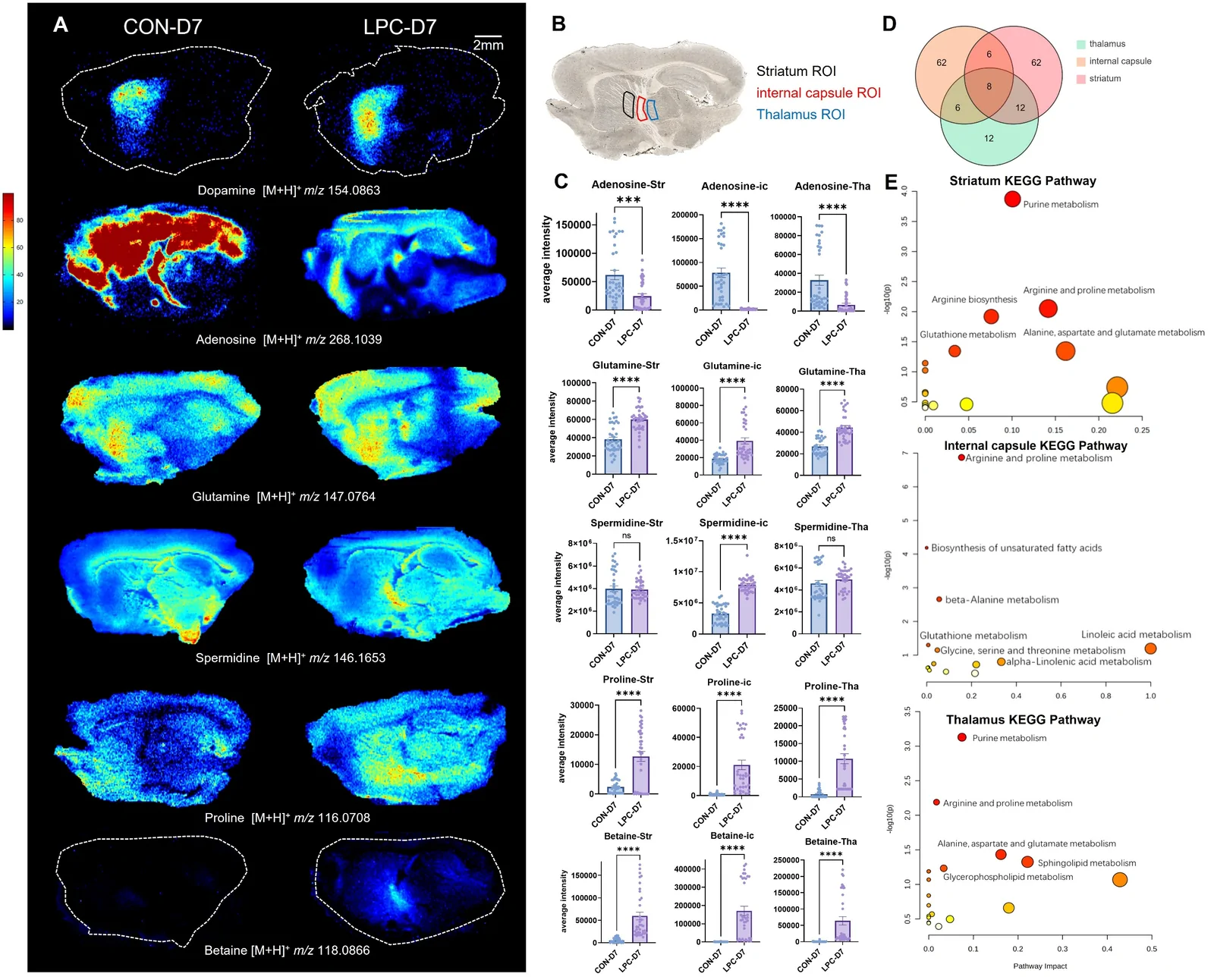
**AFADESI showed neuroactive substances changes in various substances, with pathway enrichment in the striatum, internal capsule, and thalamus**. (**A**) Representative changes in dopamine, adenosine, glutamine, spermidine, proline, and betaine after ECS partitioning barrier disruption. CON-D7 represents the contralateral, unlesioned hemisphere of the model, which serves as the control group here. (**B**) Selection of ROIs in the striatum, internal capsule, and thalamus. (**C**) Statistical results of the substances corresponding to (A) in the striatum, internal capsule, and thalamus. Data analyzed with independent sample t-tests. ****P<0.0001; ***P<0.001. (**D**) Venn diagram of differential metabolites in the three regions, showing both similarities and differences in altered substances. (**E**) KEGG pathway enrichment results for differential metabolites in the three regions.

Further metabolic pathway enrichment analysis of differential metabolites revealed significant changes in Purine metabolism and Arginine and Proline metabolism pathways in all three regions. The mass spectrometry imaging of typical differential metabolites is shown in Fig. 4A, with adenosine decreasing in all three regions, glutamine, arginine, and proline increased across all regions. Spermidine increased only in the internal capsule, and lipid dysregulation was also observed solely in the internal capsule. Surprisingly, dopamine exhibited a distribution not only in the striatum but also toward the site of ECS partitioning barrier disruption, even extending to the thalamus—a phenomenon that was not observed in the control side (Fig. 4A).

**Figure 5.**
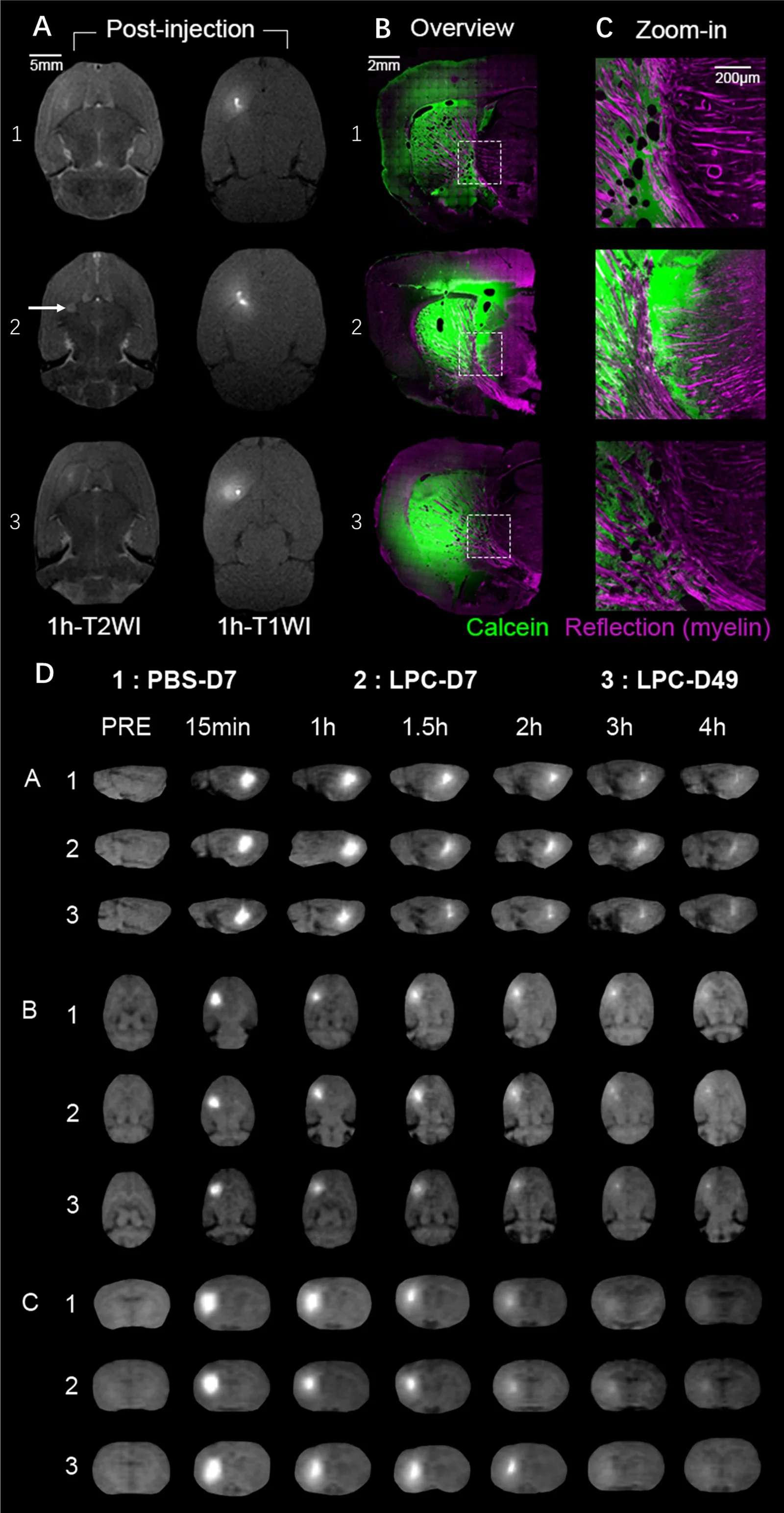
**Structural MRI, optical imaging and tracer-based MRI images demonstrated abnormal striatal drainage in the LPC-D7 group compared to the PBS-D7 (control) and LPC-D49 (recovered) groups**. (**A**) The 1h-T2WI and 1h-T1WI images show the relative scans 1 hour after stereotactic injection of Gd-DTPA injection into the striatum, with arrows indicating high signal intensity inconsistent with the normal internal capsule. By day 49, the signal at the corresponding location had returned to normal low intensity. In the LPC-D7 group, the tracer was observed crossing the internal capsule into the thalamus, a phenomenon not seen in the other groups. (**B**) Laser confocal surface imaging of the fixed sagittal sections at an oblique angle, showing the distribution of the tracer in ISF. Similar to the MRI results, tracer leakage was observed at the internal capsule in the LPC-D7 group, mainly distributed along the internal capsule. (**C**) Zoom-ins are all magnified images from the dashed box. (**D**) from top to bottom, sagittal, transverse and coronal images of the rat brain during a 4-hour tracer-based MRI procedure among rats in PBS-D7, LPC-D7, and LPC-D49. At 1 hour, the LPC-D7 group exhibited a bigger ISF diffusion range, and at 3 hours, less tracer retention in the striatum of the LPC-D7 group was observed.

### The area of abnormal ISF drainage coincided with the region’s dopamine overflowing

It is unlikely that newly generated dopaminergic neurons project to the damaged internal capsule or the adjacent thalamus. To further investigate whether ISF drainage in ECS contributed to the previously observed abnormal distribution of dopamine, we performed T2 TSE MRI at 1 hour after injection of a cocktail mixture (Gd-DTPA and optical tracer calcein) into the striatum, and LSCM surface scanning at 2 hours after injection. MRI and LSCM results collectively demonstrated that in the PBS-D7 control group, tracer diffusion from the striatum was obstructed by the intact myelin of the internal capsule, with no detectable tracer signals in the thalamus. In contrast, in the LPC-D7 group, the tracer traversed the internal capsule and reached the thalamus, consistent with the abnormal distribution of dopamine (Fig. 5A-C).

Further, we utilized tracer-based MRI to analyze ECS structure and ISF drainage changes through tracer’s disappearance within the brain striatum [19]. The images showed that while the PBS-D7 group exhibited characteristics of local metabolism within the striatum itself, the LPC-D7 group displayed massive tracer spread towards the lesion site in the internal capsule (Fig. 5D). At the initial 15-minute time point, the two groups displayed distinct drainage patterns: the tracer in the LPC-D7 group was clearly distributed towards the lesion site, forming a "droplet" shape which is similar to the dopamine distribution, while in the PBS-D7 group, the tracer appeared as a regular sphere. Over time, by the 4-hour mark, the tracer in the PBS-D7 group was almost fully drained, while residual tracer signals were still visible at the lesion site in the LPC-D7 group. Immunofluorescence staining suggested that the retention of the tracer at the lesion site may be due to increased cell density, while the increase in betaine (an osmoprotectant), as revealed by spatial metabolomics, suggests that elevated osmotic pressure at the demyelinated site may also contribute to fluid accumulation.

**Figure 6.**
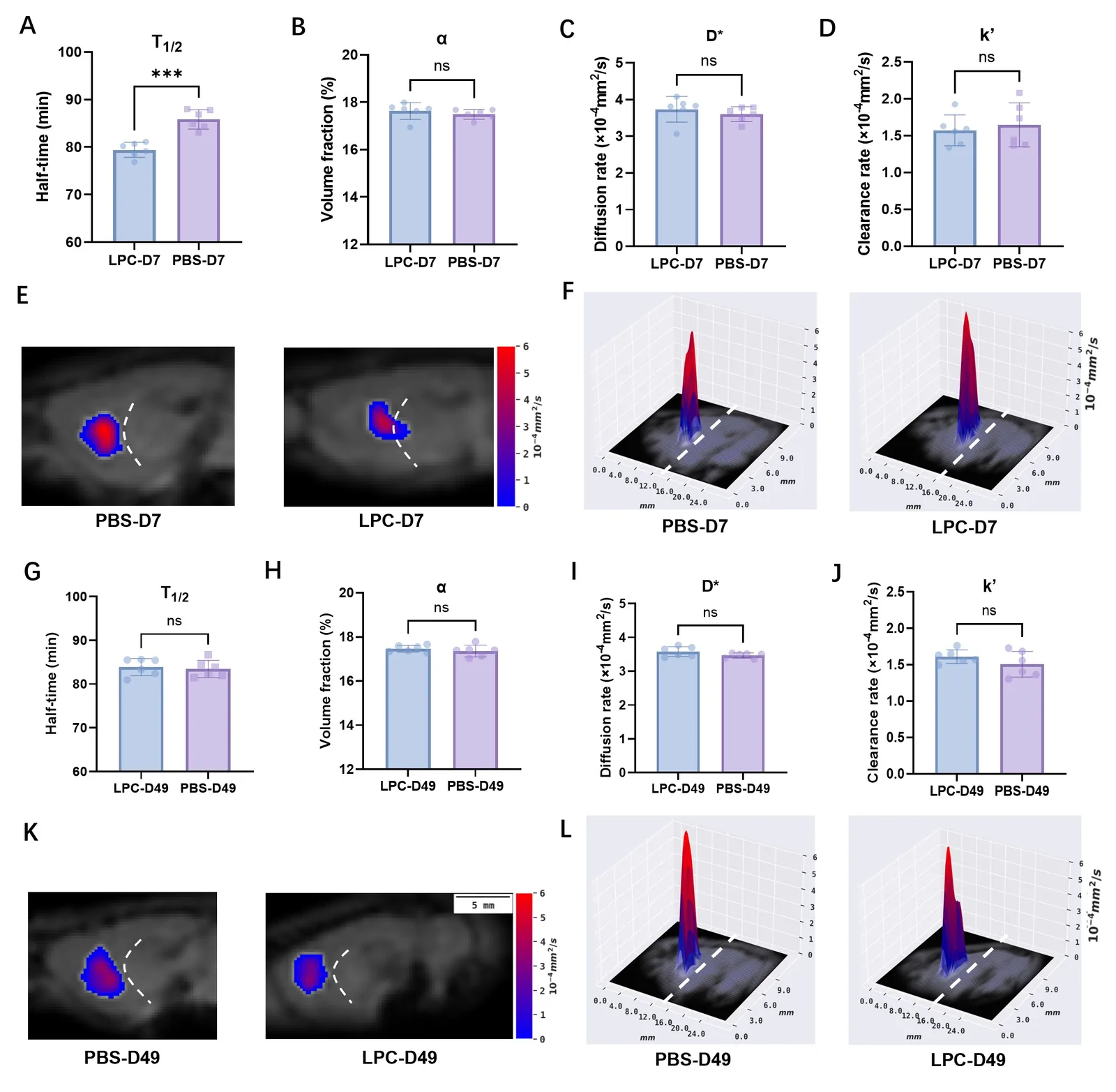
ECS and ISF drainage changes revealed by tracer-based MRI and D^*^ mapping techniques. (**A-D**) Tracer-based MRI showed a significant reduction in the half-life of ISF drainage in the striatum after the disruption of the ECS partitioning barrier in LPC-D7, although no significant differences were observed in the volume fraction, diffusion rate, or clearance rate of ECS in the striatum compared to the PBS-D7 group. (**E-F**) 2D and 3D reconstructions from D* mapping of 2 groups on day 7, which clearly illustrate that ISF in the striatum spread across the internal capsule to drain into the thalamus in the LPC-D7 group, due to the disruption of the partitioning barrier. In contrast, PBS-D7 exhibited localized metabolism within the striatum. (**G-J**) After remyelination, the striatum in the LPC-D49 group restored normal drainage half-life, with no significant difference compared to the PBS-D49 group, and no significant differences in other parameters. (**K-L**) 2D and 3D reconstructions from D* mapping of 2 groups on day 49. In (E) and (K), the dashed line represents the internal capsule, in (F) and (L), the dashed line represents the boundary toward the caudal region where the tracer was injected, showing a more posterior boundary in the LPC-D7 group compared to the other three groups. Data (A-D, G-J) analyzed with independent sample t-tests. ***P<0.001.

Regarding ECS and ISF drainage parameters, tracer-based MRI analysis revealed that the half-life of ISF drainage in the striatum was significantly shortened in the LPC-D7 group (79.42±1.58 vs. 85.81±2.03, *P*=0.0001), indicating accelerated ISF drainage in the striatum following partitioning barrier disruption in the internal capsule (Fig. 6A). Interestingly, there were no significant differences in ECS volume fraction α (LPC-D7: 17.63±0.358 vs. PBS-D7: 17.49±0.206, *P*=0.4399, Fig. 6B), effective diffusion coefficient D* (LPC-D7: 3.735±0.349 vs. PBS-D7: 3.603±0.202, *P*=0.4398, Fig. 6C), or clearance coefficient k’ (LPC-D7: 1.572±0.209 vs. PBS-D7: 1.647±0.298, *P*=0.6264, Fig. 6D), suggesting that ECS structure in the striatum were not significantly altered. D* mapping further confirmed abnormal ISF drainage in the LPC-D7 group, with altered diffusion coefficients observed in both the internal capsule and thalamus (Fig. 6E, F). These results indicated that abnormal ISF drainage areas and rates occurred in the striatum.

To further investigate holistic drainage route of striatum ISF, we injected ^99m^Tc-DTPA into the unilateral striatum of normal adult rats. The results showed that the tracer’s trajectory extensively spread to the ipsilateral cortex but was confined to the ipsilateral hemisphere, without reaching the contralateral side (Supplementary Fig.3).

### Reversed striatum ISF drainage and dopamine distribution after remyelination

Recognizing that animals have the capacity to restore their motor abilities, we investigated whether the abnormal drainage could recover following remyelination. We conducted continuous T2-weighted TSE structural imaging of the same lesioned animals across multiple days. Contrast-enhanced MRI demonstrated that complete recovery at the lesion site was not observed until day 49 (Supplementary Fig. 4). Furthermore, we conducted optical imaging validation and histological staining, and the LPC-D49 group showed results similar to those of the PBS-D7 group, suggesting near-complete remyelination (Fig. 5A-C).

We observed recovered, normal ISF drainage on day 49 after remyelination. Tracer-based MRI analysis demonstrated that tracer distribution was confined to the striatum itself, with the half-life of striatal ISF drainage showing no significant difference between the LPC-D49 and PBS-D49 groups (83.89±1.96 min vs. 83.44±1.97 min, P=0.7036, Fig. 6G). This suggests that ISF drainage in the striatum returned to its normal range and rate following myelin regeneration. Additionally, there was no significant difference in striatal ECS volume fraction α, effective diffusion coefficient D*, and clearance coefficient k’ (Fig. 6H-J). The D* mapping results demonstrated that the LPC-D49 group restored the diffusion coefficient characteristics unique to the striatum, matching those of both PBS-D7 and PBS-D49 groups (Fig. 6K, L).

To further validate our hypothesis, we analyzed the spatial metabolomics results from the remyelination group (LPC-D49) and the long-term recovery group (LPC-D147), which were derived from the remaining animals in the behavioral test groups. As anticipated, dopamine distribution in both the remyelination and long-term recovery groups normalized, with no evidence of abnormal cross-regional distribution. Moreover, glutamine showed signs of recovery, whereas adenosine did not fully return to normal levels and seemed to remain at levels consistent with those observed in the demyelinated state (Supplementary Fig. 5). These findings may warrant further investigation due to the limited sample size. Overall, these findings suggest that specific disturbances in neuroactive substances in this model may be closely associated with abnormalities in ISF drainage.

## DISCUSSION

Our study suggests that abnormal ISF drainage may represent a novel mechanism underlying neurochemical imbalance in MS, providing valuable insights into its pathogenesis and neuropsychiatric symptoms. In addition to the previously recognized adverse effects associated with changes in the levels of neuroactive substances, our findings also highlight that the specific distribution of these substances may contribute to the neurochemical imbalance. This new perspective offers a deeper understanding of the neurobiology of MS and opens potential avenues for the development of targeted therapeutic strategies.

To our knowledge, this is the first report of spatial metabolomics profiling following localized demyelination induced by LPC. Although myelin disruption occurred solely in the internal capsule, alterations in numerous metabolites were observed throughout the brain, including key neurotransmitters, neuromodulators, amino acids, and lipids. Among these substances, dopamine was particularly prominent. As a key excitatory neurotransmitter in the brain, dopamine typically exhibits region-specific distribution, with high concentrations in the striatum and minimal presence in the thalamus in the normal brain, as demonstrated by a modified matrix-assisted laser desorption/ionization mass spectrometry imaging (MALDI-MSI) method [20]. However, in our model, we observed dopamine distribution extending from the striatum towards the lesion site and even into the thalamus, suggesting an expanded range of volume transmission.

**Figure 7.**
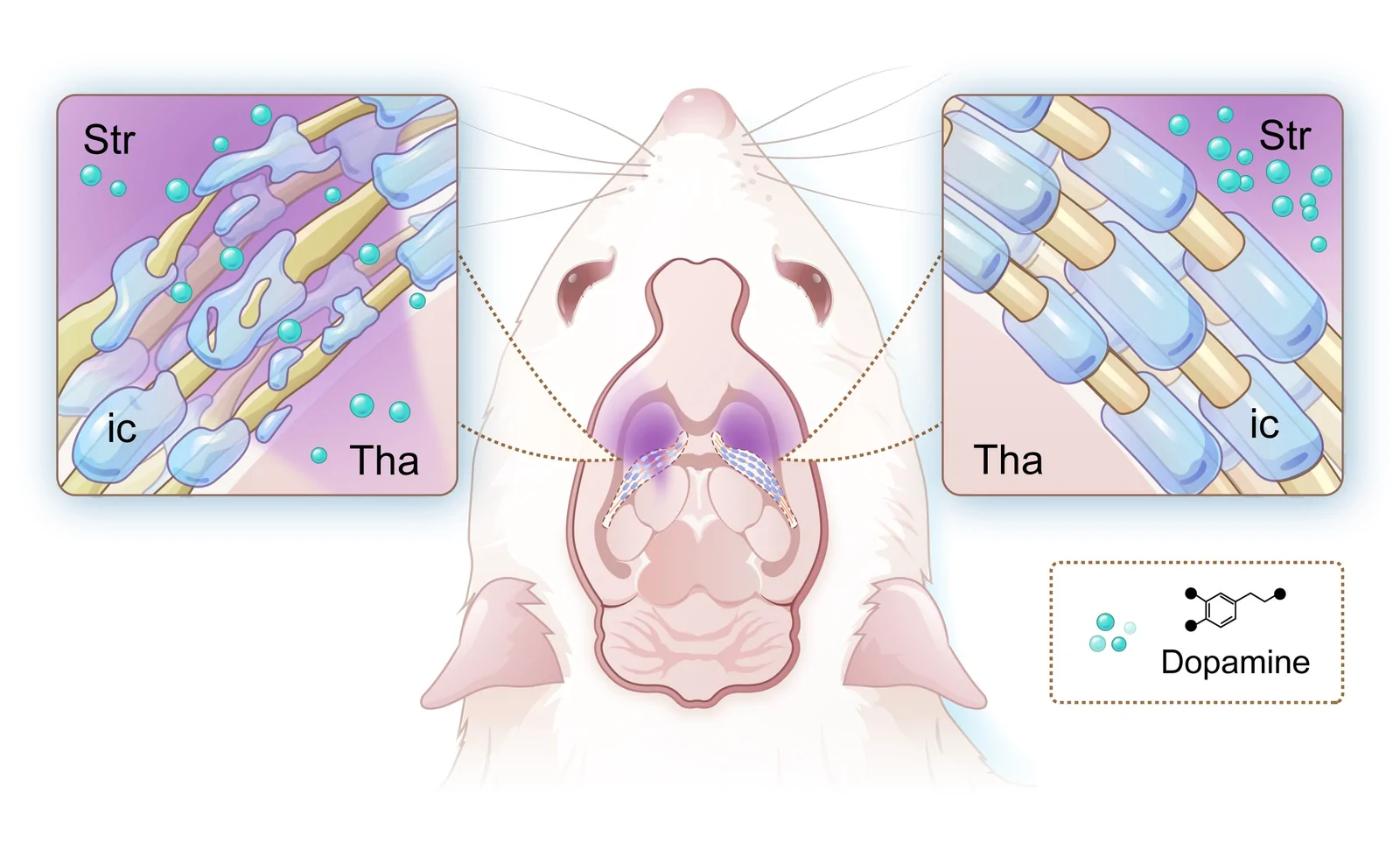
**Illustration of a novel mechanism for neurochemical imbalance: abnormal ISF drainage resulting from focal ECS barrier disruption**. LPC was injected into the left internal capsule, resulting in noticeable demyelination by day 7. Demyelination within the internal capsule (ic) disrupted the ISF drainage from the striatum (Str) to the thalamus (Tha), leading to dopamine leakage (represented by light blue spheres) from the striatum into the thalamus. On the contralateral side, healthy myelinated fibers (yellow-blue) are shown, with normal ISF drainage and dopamine distribution. The violet-purple color highlights the ISF drainage route from the striatum.

Using tracer-based MRI, we observed that the tracer injected into the striatum drained into the adjacent thalamus in focal demyelinated animals, similar to findings in previous studies using the cuprizone model [5]. The abnormal dopamine distribution revealed by the spatial metabolomics results was consistent with the reflux pattern of ISF drainage (as illustrated in Fig. 7). The D* mapping technique, which involved long-term continuous dynamic scanning of tracer dissipation, generated detailed two- and three-dimensional diffusion parameter maps. These maps revealed cross-regional tracer drainage patterns that also aligned with the abnormal dopamine distribution. Accelerated clearance was observed in the striatum of the demyelination group; however, no alterations were found in the structural characteristics of the striatal ECS. This accelerated drainage may be attributed to the tracer reaching the thalamus, which exhibits a faster drainage rate compared to the striatum [21]. As spontaneous remyelination occurred, the range of ISF drainage gradually returned to normal, indicating the restoration of proper interstitial fluid dynamics. Concurrently, the balance of neuroactive substances was re-established, suggesting a recovery of neurochemical homeostasis. The results from recovery experiments provided positive evidence supporting these observations, demonstrating that the repair of ECS barriers through remyelination not only normalized ISF drainage pathways but also contributed to the stabilization of neuroactive substances distribution across affected regions. We also observed extensive drainage only in the ipsilateral hemisphere following unilateral injection of the radioactive tracer in SPECT/CT, which was consistent with results from fluorescence tracer [22]. As metabolites changed drastically across the entire injected hemisphere, similar to the ISF drainage area observed in the ipsilateral half, we hypothesize that the imbalance of substances between the two hemispheres may result from ISF diffusion.

Dopamine dysregulation in the thalamus has been implicated as a potential contributor to neuropsychiatric symptoms, such as schizophrenia [23], which are frequently observed in patients with MS [24]. The thalamic reticular nucleus (TRN), a GABAergic structure located adjacent to the striatum and internal capsule, lacks intrinsic dopamine but exhibits one of the highest densities of D4 dopamine receptors among subcortical regions [25]. Research indicates that dopamine enhances the spontaneous basal firing of TRN neurons [26], and elevated thalamic DA levels have been shown to induce bursts of delta waves, potentially creating a positive feedback loop [27]. This heightened electrical activity is hypothesized to play a role in the pathophysiology of schizophrenia. Supporting this notion, clinical studies have reported increased dopamine levels in the thalamus of patients with schizophrenia [28], suggesting a potential link between elevated DA levels and neurochemical dysfunctions.

Recent findings also provide indirect evidence linking elevated thalamic DA levels to depressive-like behaviors, potentially mediated by their effects on the TRN. For instance, optogenetic studies have demonstrated that the inhibition of the TRN to the lateral habenula circuit induces depressive-like behaviors [29], highlighting the TRN's role in mood regulation. Furthermore, electrophysiological evidence suggests that DA suppresses TRN activity by inhibiting neuronal firing through presynaptic D4 receptor-mediated modulation [30]. These findings imply that elevated DA levels in the thalamus may disrupt TRN function, contributing to depressive-like symptoms in neuropsychiatric conditions.

In addition to neuroactive substances dysregulation, structural changes in white matter microstructure are associated with neurocognitive impairments. Studies have shown that reduced white matter integrity may lead to compensatory increases in white matter connectivity in patients with cognitive impairments [31]. For example, cognitive fatigue has been linked to decreased fractional anisotropy in the anterior internal capsule, correlating with higher self-reported fatigue scores [32]. These findings align with the hypothesis that microstructural alterations in white matter contribute to cognitive and neuropsychiatric symptoms in MS.

The enrichment of purine and arginine-proline metabolic pathways observed in our study suggests their potential involvement in MS pathophysiology. Previous review has highlighted altered purine metabolism in MS, emphasizing its role in inflammation, oxidative stress, and apoptosis—key processes in MS progression [33]. Similarly, arginine metabolism, particularly through nitric oxide synthesis, impacts neuroinflammation and vascular dysfunction, both central to MS pathogenesis [34]. Although the link between arginine-proline metabolism and MS is less direct, these pathways may contribute to neuroinflammation and neuronal damage. Metabolic shifts in these pathways suggest a potential connection to MS, yet it remains unclear whether they directly increase MS risk or are consequences of disease progression. Experimental validation is essential to determine their roles in MS onset and progression. Moreover, the interplay between neuroinflammation and metabolic changes complicates the interpretation of the observed results, making it challenging to discern their primary drivers. Future research should aim to disentangle the effects of ECS alterations from those of neuroinflammation to clarify the mechanisms behind the observed metabolic imbalance in MS. Further investigation is also needed to determine whether ECS alterations play a causal role in these processes.

## Supplementary Materials

The Supplementary data can be found online at: www.aginganddisease.org/EN/10.14336/AD.2024.1444.
