# Supplementary material for: New Insight into the Mechanism of Neurochemical Imbalance in Multiple Sclerosis: Abnormal Transportation of Brain Extracellular Space
Source: Aging Dis. 2025 Feb 11;17(1):452–65. doi: 10.14336/AD.2024.1444 (PMC12727071; doi:10.14336/AD.2024.1444)
Supplement: Supplementary file 1 — The Supplementary data can be found online at: www.aginganddisease.org/EN/10.14336/AD.2024.1444. [file AD-17-1-452-s.pdf]

SUPPLEMENTARY DATA

New Insight into the Mechanism of Neurochemical Imbalance in Multiple Sclerosis: Abnormal Transportation of Brain Extracellular Space

Yumeng Cheng, Jiao Liu, Feng Tian, Hanbo Tan, Tianyu Wang, Jiabin Lu, Zeqing Tang, Xinlei Ma, Jingge Lian, Shaoyi Su, Yu Fu, Bin Liu, Yuliang Li, Wanyi Fu, Meng Xu, Hongbin Han

© 2024. Cheng Y et al. Published online at http://www.aginganddisease.org/EN/10.14336/AD.2024.1444

SUPPLEMENTARY DATA

Supplementary Table 1. AFADESI-MSI acquisition parameters

Parameters	Settings
Polarity	Positive Ion/Negative Ion
Scan mode	Full Scan
Scan range	70~1000 Da
AGC time	200 ms
AGC target	3e6
Resolution	70,000

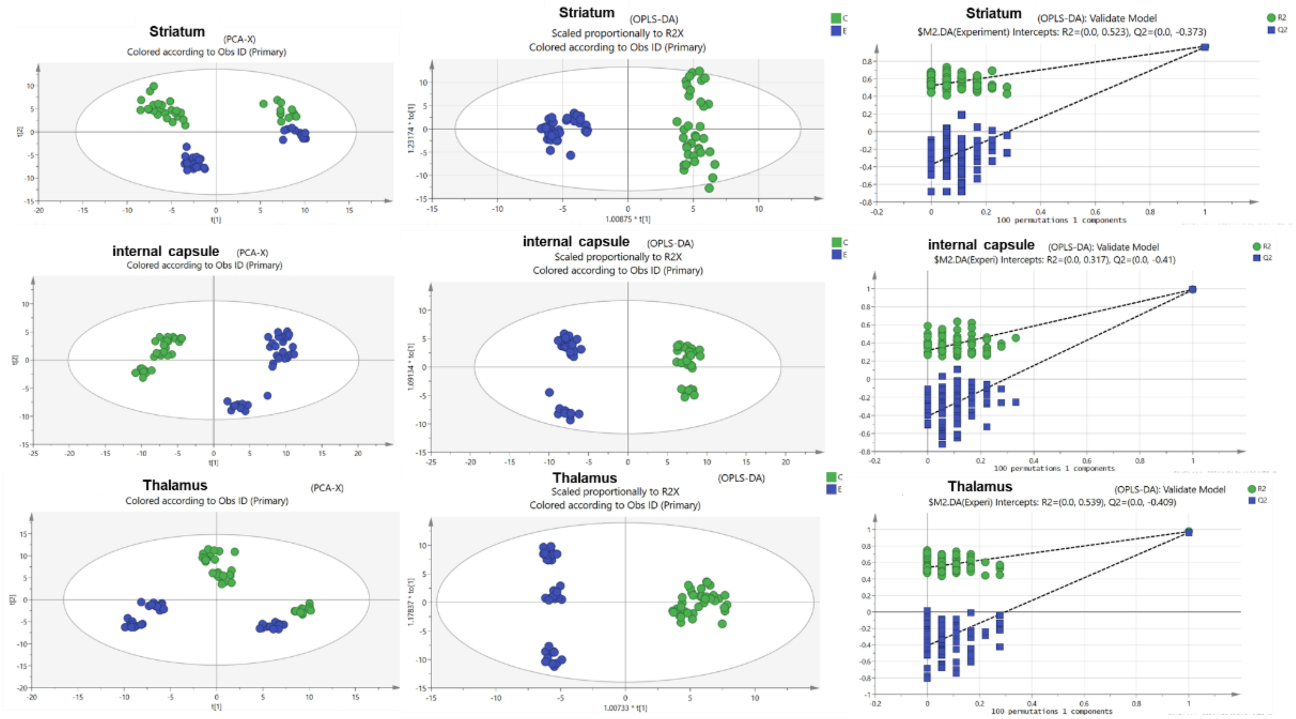

Supplementary Figure 1. Spatial metabolomics data from the striatum, internal capsule, and thalamus regions under positive ion mode. The PCA analysis and supervised OPLS-DA revealed a certain clustering and natural grouping trend of metabolites between the experimental and control groups in the three regions. Permutation testing was used to validate the model's effectiveness. After 100 rounds of modeling, the regression line formed by R2Y (green dots) and the R2Y values of the actual model indicated that the model was relatively robust.

© 2024. Cheng Y et al. Published online at http://www.aginganddisease.org/EN/10.14336/AD.2024.1444

SUPPLEMENTARY DATA

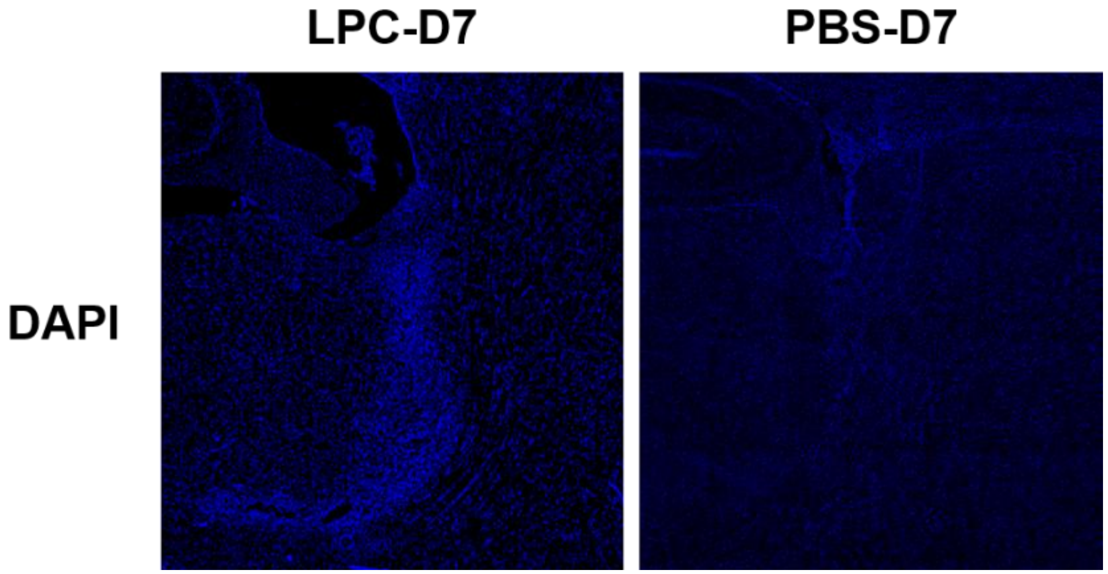

Supplementary Figure 2. DAPI signal enriched in demyelination cite. This phenomenon indicates that accumulation of tracers in demyelination sites may be related with increased cell intensity.

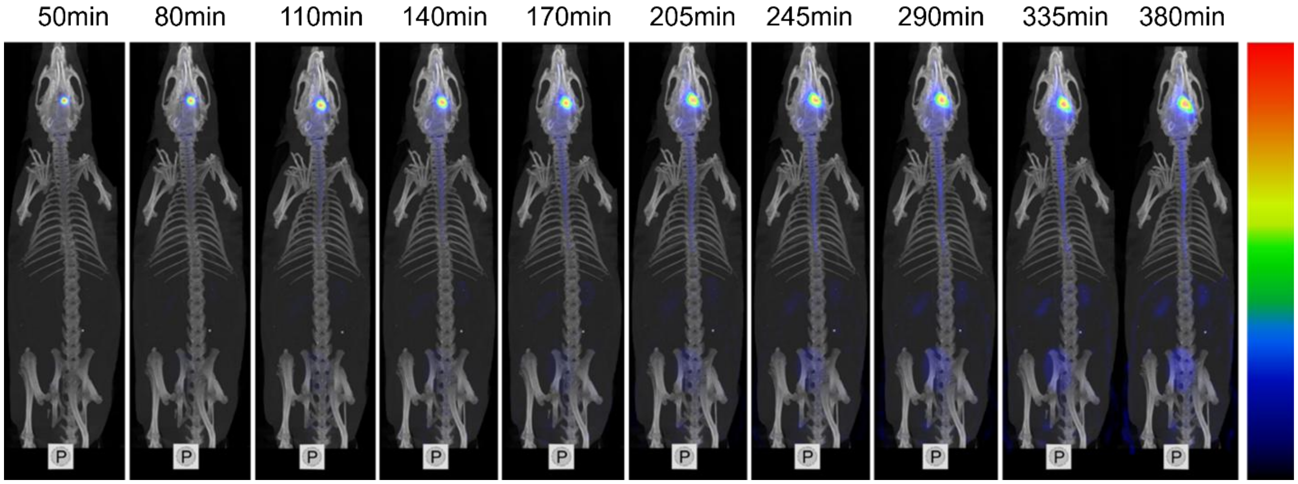

Supplementary Figure 3. The drainage of 99mTc-DTPA was confined to the ipsilateral hemisphere following unilateral injection into the striatum. The time points for each image are indicated above.

© 2024. Cheng Y et al. Published online at http://www.aginganddisease.org/EN/10.14336/AD.2024.1444

SUPPLEMENTARY DATA

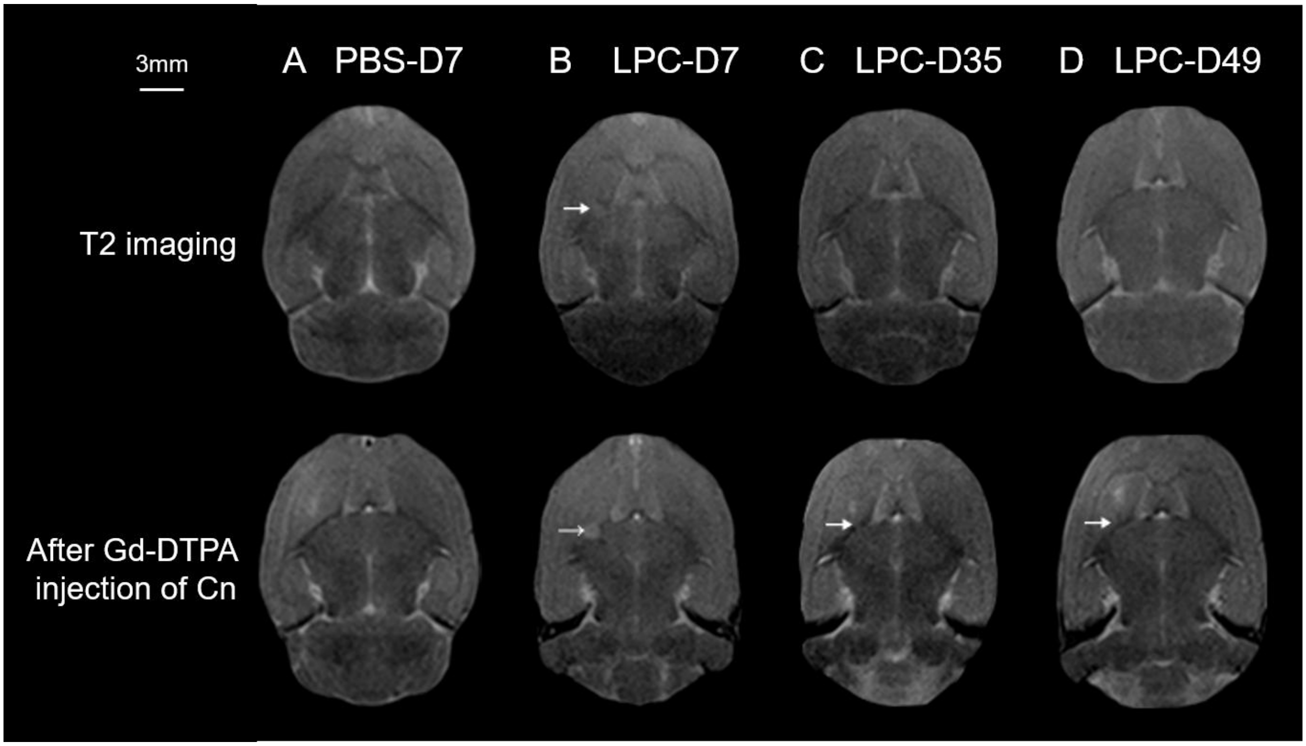

Supplementary Figure 4. Myelin of internal capsule did not recover completely until day 49 after LPC injection. (A) The MRI T2 TSE image of the PBS-D7 group and 1 hour after tracer injection into the striatum. (B), (C), and (D) show the corresponding images of LPC-D7, LPC-D35, and LPC-D49. On day 35, it is difficult to identify remyelination extent from the single T2 structural scan; however, after Gd-DTPA injection into the striatum, enhancement at the internal capsule could be seen, suggesting uncomplete recovery of the myelin. The arrows indicate the lesion site. The upper and lower rows in the figure correspond to the same animal.

© 2024. Cheng Y et al. Published online at http://www.aginganddisease.org/EN/10.14336/AD.2024.1444

SUPPLEMENTARY DATA

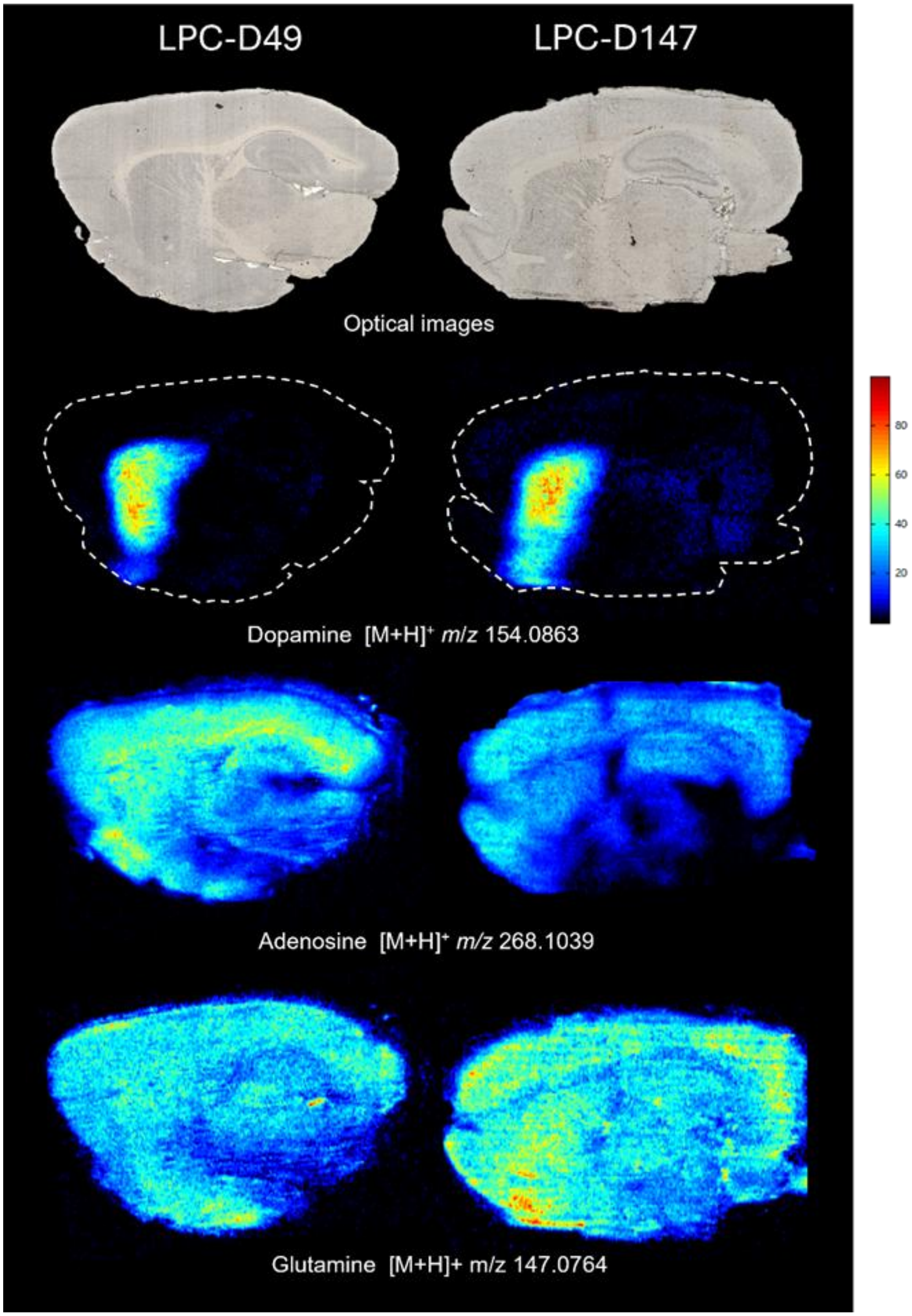

Supplementary Figure 5. Spatial metabolomics results of remyelination and long-term recovery group of our model. Dopamine, adenosine and glutamine returned similar signal intensity with control animals (CON-D7).

© 2024. Cheng Y et al. Published online at http://www.aginganddisease.org/EN/10.14336/AD.2024.1444

SUPPLEMENTARY DATA

Supplementary Table 2. 88 differential metabolites in striatum in LPC-D7.

m/z	Compound id	Compound name	formula	Monoisotopic mass	Adduct	Adduct type	FC	Changes
116.0709	HMDB0000162	Proline	C5H9NO2	115.0633	M+H	+	5.275024	UP
118.0866	HMDB0000043	Betaine	C5H12NO2	118.0868	M+H	+	11.03215	UP
130.0501	HMDB0000267	Pyroglutamic acid	C5H7NO3	129.0426	M+H	+	1.442909	UP
136.0619	HMDB0000034	Adenine	C5H5N5	135.0545	M+H	+	0.055916	DOWN
137.0459	HMDB0000157	Hypoxanthine	C5H4N4O	136.0385	M+H	+	1.820158	UP
147.0764	HMDB0000641	Glutamine	C5H10N2O3	146.0691	M+H	+	1.573266	UP
156.0422	HMDB0000883	L-Valine	C5H11NO2	117.079	M+K	+	1.648314	UP
156.077	HMDB0000177	Histidine	C6H9N3O2	155.0695	M+H	+	24.6046	UP
156.1496	HMDB0244825	1-Ethyl-3-(3-dimethylaminopropyl)carbodiimide	C8H17N3	155.1422	M+H	+	2.904272	UP
158.0403	HMDB0304083	2-oxo-6-methylthiohexanoate	C7H11O3S	175.0434	M+H-H2O	+	2.645926	UP
184.0733	HMDB0001565	Phosphorylcholine	C5H15NO4P	184.0739	M+H	+	1.50848	UP
188.1758	HMDB0001276	N1-Acetylspermidine	C9H21N3O	187.1685	M+H	+	2.063891	UP
197.1011	HMDB0000517	L-Arginine	C6H14N4O2	174.1117	M+Na	+	3.665461	UP
204.1232	HMDB0000201	L-Acetylcarnitine	C9H17NO4	203.1158	M+H	+	11.09121	UP
218.0965	HMDB0041793	1-Aminopyrene	C16H11N	217.0891	M+H	+	0.314517	DOWN
229.1546	HMDB0304810	Pro-Ile	C11H20N2O3	228.1474	M+H	+	2.040953	UP
241.1294	HMDB0000745	Homocarnosine	C10H16N4O3	240.1222	M+H	+	0.547768	DOWN
241.1546	HMDB0015407	Pirbuterol	C12H20N2O3	240.1474	M+H	+	1.493914	UP
249.1557	HMDB0244416	2-[(Z)-[(4Z)-4-(Diaminomethylidenehydrazinylidene)hexan-3-ylidene]amino]guanidine	C8H18N8	226.1654	M+Na	+	3.029216	UP
268.1039	HMDB0000050	Adenosine	C10H13N5O4	267.0968	M+H	+	0.398042	DOWN
283.0942	HMDB0032472	Polyethylene, oxidized	C12H20O5	244.1311	M+K	+	1.652053	UP
367.2033	HMDB0002183	Docosahexaenoic acid	C22H32O2	328.2402	M+K	+	1.773387	UP
383.2036	HMDB0258626	Sumarotene	C24H30O2S	382.1967	M+H	+	2.760666	UP
385.1022	HMDB0033442	Mytilin B	C14H22N2O8	346.1376	M+K	+	1.857318	UP
401.2662	HMDB0011579	MG(20:4(8Z,11Z,14Z,17Z)/0:0/0:0)	C23H38O4	378.277	M+Na	+	0.473492	DOWN
441.2402	HMDB0011587	MG(22:6(4Z,7Z,10Z,13Z,16Z,19Z)/0:0/0:0)	C25H38O4	402.277	M+K	+	0.500006	DOWN
458.3111	HMDB0257493	Sarar	C21H41N9	419.3485	M+K	+	2.98135	UP
476.2339	HMDB0247839	2-Chloro-1-[(2S,3S,5S,10S,13S)-3-hydroxy-10,13-dimethyl-2-morpholino-2,3,4,5,6,7,8,9,11,12,14,15,16,17-tetradecahydro-1H-cyclopenta[a]phenanthren-17-yl]ethanone	C25H40ClNO3	437.2697	M+K	+	2.104721	UP
604.5065	HMDB0004950	Cer(d18:1/18:0)	C36H71NO3	565.5434	M+K	+	1.696115	UP
766.514	HMDB0011440	PE(P-18:1(9Z)/18:1(11Z))	C41H78NO7P	727.5516	M+K	+	0.530804	DOWN
768.5873	HMDB0013412	PC(O-34:1)	C42H84NO7P	745.5985	M+Na	+	0.544141	DOWN
772.62	HMDB0011307	PC(P-18:1(9Z)/18:0)	C44H86NO7P	771.6142	M+H	+	0.585181	DOWN
808.5823	HMDB0000593	PC(36:2)	C44H84NO8P	785.5935	M+Na	+	0.674476	DOWN
848.6354	HMDB0004975	GlcCer(d18:1/24:1)	C48H91NO8	809.6745	M+K	+	0.386004	DOWN
852.5882	HMDB0008045	PC(18:0/20:2(11Z,14Z))	C46H88NO8P	813.6248	M+K	+	2.988923	UP
866.6462	HMDB0252577	Galactocerebrosides	C48H93NO9	827.685	M+K	+	0.502515	DOWN
125.0956	HMDB0304443	octanoate	C8H15O2	143.1072	M-H20-H	-	0.031382	DOWN
136.0152	HMDB0246202	Cytosine-5-carboxylic acid	C5H5N3O3	155.0331	M-H20-H	-	0.036301	DOWN
185.0446	HMDB0041031	3,5,6-Trihydroxy-5-(hydroxymethyl)-2-methoxy-2-cyclohexen-1-one	C8H12O6	204.0634	M-H20-H	-	0.64977	DOWN
185.1536	HMDB0245961	1,2-Propanediol, 3-(octyloxy)-	C11H24O3	204.1725	M-H20-H	-	0.066819	DOWN
199.1329	HMDB0254850	2,3-Dihydroxypropyl octanoate	C11H22O4	218.1518	M-H20-H	-	0.051756	DOWN
211.0967	HMDB0059744	3,4-Methyleneazelaic acid	C11H16O4	212.1049	M-H	-	2.083268	UP
213.0155	HMDB0249913	Polyribosylribitolphosphate	C5H13O8P	232.0348	M-H20-H	-	0.045559	DOWN
213.076	HMDB0031640	Glycerol 1-propanoate diacetate	C10H16O6	232.0947	M-H20-H	-	0.618346	DOWN
216.9181	HMDB0060490	Methylselenopyruvate	C4H6O3Se	181.9482	M+Cl	-	0.381417	DOWN
217.0709	HMDB0029592	Triacetin	C9H14O6	218.079	M-H	-	0.576677	DOWN

© 2024. Cheng Y et al. Published online at http://www.aginganddisease.org/EN/10.14336/AD.2024.1444

226.035	HMDB0006794	5-(2'-Carboxyethyl)-4,6-Dihydroxypicolinate	C9H9NO6	227.043	M-H	-	0.448613	DOWN
232.9438	HMDB0246204	1,1,1-Trifluoro-2,4-pentanedione	C5H5F3O2	154.0242	M+Br	-	0.471537	DOWN
242.0276	HMDB0035996	2-(Methylthio)-3H-phenoxazin-3-one	C13H9NO2S	243.0354	M-H	-	0.673306	DOWN
244.137	HMDB0059773	S-3-oxodecanoyl cysteamine	C12H23NO2S	245.1449	M-H	-	2.662457	UP
250.1442	HMDB0245262	2-Nitrophenyl octyl ether	C14H21NO3	251.1521	M-H	-	0.067317	DOWN
251.1284	HMDB0036047	3alpha-Hydroxyoreadone	C14H20O4	252.1362	M-H	-	2.476154	UP
253.2168	HMDB0003229	Palmitoleic acid	C16H30O2	254.2246	M-H	-	0.137248	DOWN
264.0098	HMDB0245169	2-Hydroxysaclofen	C9H12ClNO4S	265.0176	M-H	-	0.615632	DOWN
282.0839	HMDB0000133	Guanosine	C10H13N5O5	283.0917	M-H	-	0.178737	DOWN
289.0372	HMDB0240369	2-Acetylaminophenoxazin-3-one	C14H10N2O3	254.0691	M+Cl	-	0.752775	DOWN
294.0287	HMDB0243786	1-(3-Carboxypropylcarbamoyl)-5-fluorouracil	C9H10FN3O5	259.0604	M+Cl	-	3.986213	UP
303.083	HMDB0001067	N-Acetylaspartylglutamic acid	C11H16N2O8	304.0907	M-H	-	0.313738	DOWN
313.0898	HMDB0259163	Tribufos	C12H27OPS3	314.0962	M-H	-	2.891116	UP
324.986	HMDB0257416	S-Nitrosocaptopril	C9H14N2O4S	246.0674	M+Br	-	0.340734	DOWN
325.1252	HMDB0032223	Diisopentyl thiomalate	C14H26O4S	290.1552	M+Cl	-	1.915335	UP
327.0091	HMDB0258570	Sulcotrione	C14H13ClO5S	328.0172	M-H	-	0.462121	DOWN
333.9379	HMDB0250495	4-Methyl-2-oxo-2H-chromen-7-yl sulfamate	C10H9NO5S	255.0201	M+Br	-	8.089146	UP
335.2223	HMDB0002982	Prostaglandin B1	C20H32O4	336.2301	M-H	-	1.653835	UP
359.9761	HMDB0246385	4-Chloro-3-ethoxy-7-guanidinoisocoumarin	C12H12ClN3O3	281.0567	M+Br	-	0.403189	DOWN
360.9612	HMDB0252116	Etoprine	C12H12Cl2N4	282.0439	M+Br	-	1.720697	UP
362.0503	HMDB0001397	Guanosine monophosphate	C10H14N5O8P	363.058	M-H	-	8.924042	UP
364.9375	HMDB0258577	Sulfametrole	C9H10N4O3S2	286.0194	M+Br	-	0.513476	DOWN
365.3421	HMDB0002368	Nervonic acid	C24H46O2	366.3498	M-H	-	0.722901	DOWN
380.0821	HMDB0255991	Oxo-ciprofloxacin	C17H16FN3O4	345.1125	M+Cl	-	3.619301	UP
389.9954	HMDB0246929	(S)-5-[(4-Amino-4-carboxy-1-oxobutyl)amino]-2-nitrobenzoic acid	C12H13N3O7	311.0753	M+Br	-	0.449135	DOWN
399.0219	HMDB0041463	2,3-Dihydro-2,3-dihydroxy-4-(4-methoxyphenyl)-1H-phenalen-1-one	C20H16O4	320.1049	M+Br	-	0.075835	DOWN
399.2021	HMDB0036855	Cincassiol B	C20H32O8	400.2097	M-H	-	1.735028	UP
401.0015	HMDB0037110	4-(3,4-Dihydroxyphenyl)-2,3-dihydro-2,3-dihydroxy-1H-phenalen-1-one	C19H14O5	322.0841	M+Br	-	0.524741	DOWN
405.9462	HMDB0252247	Fiacitabine	C9H11FIN3O4	370.9778	M+Cl	-	0.225292	DOWN
417.0148	HMDB0252798	Glufosfamide	C10H21Cl2N2O7P	382.0463	M+Cl	-	0.257327	DOWN
421.9429	HMDB0013991	5'-Hydroxylornoxicam	C13H10ClN3O5S2	386.975	M+Cl	-	0.139923	DOWN
423.9568	HMDB0248806	Ethyl azinphos	C12H16N3O3PS2	345.0371	M+Br	-	0.374578	DOWN
432.9832	HMDB0302901	Flavogallol	C21H8O12	452.0016	M-H20-H	-	0.364986	DOWN
434.0215	HMDB0014809	Cefixime	C16H15N5O7S2	453.0413	M-H20-H	-	0.270826	DOWN
438.974	HMDB0247515	4-Hydroxy-3-(2'-hydroxy-[1,1'-biphenyl]-4-yl)-6-oxo-6,7-dihydrothieno[2,3-b]pyridine-5-carbonitrile	C20H12N2O3S	360.0569	M+Br	-	0.308814	DOWN
450.9756	HMDB0254800	1H-1,4-Diazepine, hexahydro-1-((5-iodo-1-naphthalenyl)sulfonyl)-	C15H17IN2O2S	416.0055	M+Cl	-	0.389431	DOWN
598.4972	HMDB0011774	Cer(d18:1/18:1(11Z))	C36H69NO3	563.5277	M+Cl	-	1.821666	UP
611.1443	HMDB0003337	Oxidized glutathione	C20H32N6O12S2	612.152	M-H	-	0.454142	DOWN
716.5229	HMDB0008926	PE(16:0/18:1(11Z))	C39H76NO8P	717.5309	M-H	-	1.748383	UP
760.5131	HMDB0112267	PS(16:0/18:1(11Z))	C40H76NO10P	761.5207	M-H	-	2.319035	UP
808.5111	HMDB0112415	PS(18:1(9Z)/20:4(8Z,11Z,14Z,17Z))	C44H76NO10P	809.5207	M-H	-	2.372957	UP
818.5329	HMDB0112383	PS(18:0/22:5(4Z,7Z,10Z,13Z,16Z))	C46H80NO10P	837.552	M-H20-H	-	2.558324	UP

Supplementary Table 3. 82 differential metabolites in internal capsule in LPC-D7.

m/z	Compound id	Compound name	formula	Monoisotopic mass	adduct	Adduct type	FC	Changes
116.0708	HMDB0000162	Proline	C5H9NO2	115.0633285	M+H	+	36.59887299	UP

118.0866	HMDB0000043	Betaine	C5H12NO2	118.0868036	M+H	+	170.9173028	UP
126.0528	HMDB0000112	gamma-Aminobutyric acid	C4H9NO2	103.0633285	M+Na	+	0.442884976	DOWN
146.0926	HMDB0003464	4-Guanidinobutanoic acid	C5H11N3O2	145.0851266	M+H	+	0.652846125	DOWN
146.1653	HMDB0001257	Spermidine	C7H19N3	145.1578976	M+H	+	2.429464553	UP
152.0222	HMDB0000562	Creatinine	C4H7N3O	113.0589119	M+K	+	0.591746485	DOWN
156.0769	HMDB0000177	Histidine	C6H9N3O2	155.0694765	M+H	+	19.04187459	UP
161.1286	HMDB0002038	N(6)-Methyllysine	C7H16N2O2	160.1211778	M+H	+	0.335502263	DOWN
170.0328	HMDB0000064	Creatine	C4H9N3O2	131.0694765	M+K	+	0.529465692	DOWN
188.1758	HMDB0001276	N1-Acetylspermidine	C9H21N3O	187.1684623	M+H	+	12.13474536	UP
203.2231	HMDB0001256	Spermine	C10H26N4	202.2157469	M+H	+	3.976010535	UP
218.1865	HMDB0060066	Spermic acid 1	C10H23N3O2	217.179027	M+H	+	0.207079542	DOWN
241.1293	HMDB0000745	Homocarnosine	C10H16N4O3	240.1222404	M+H	+	0.161376505	DOWN
268.104	HMDB0000050	Adenosine	C10H13N5O4	267.0967539	M+H	+	0.023051395	DOWN
291.0701	HMDB0000195	Inosine	C10H12N4O5	268.0807695	M+Na	+	0.435093352	DOWN
297.1462	HMDB0000872	Tetradecanedioic acid	C14H26O4	258.1831093	M+K	+	0.552153012	DOWN
313.2139	HMDB0002961	Dihydrotestosterone	C19H30O2	290.2245802	M+Na	+	0.442214973	DOWN
323.1619	HMDB0000010	2-Methoxyestrone	C19H24O3	300.1725446	M+Na	+	0.433248889	DOWN
325.1773	HMDB0000672	Hexadecanedioic acid	C16H30O4	286.2144094	M+K	+	0.485592461	DOWN
344.2792	HMDB0241932	N-Palmitoyl Serine	C19H37NO4	343.2722587	M+H	+	0.399197651	DOWN
367.2033	HMDB0002183	Docosahexaenoic acid	C22H32O2	328.2402303	M+K	+	6.084665162	UP
370.2953	HMDB0242185	N-Oleoyl-L-Serine	C21H39NO4	369.2879087	M+H	+	0.31462973	DOWN
398.3267	HMDB0006317	trans-Hexadec-2-enoyl carnitine	C23H43NO4	397.3192089	M+H	+	0.44034105	DOWN
401.2662	HMDB0011579	MG(20:4(8Z,11Z,14Z,17Z)/0:0/0:0)	C23H38O4	378.2770097	M+Na	+	0.265413135	DOWN
496.3397	HMDB0010382	LysoPC(16:0/0:0)	C24H50NO7P	495.3324895	M+H	+	3.632065106	UP
546.3525	HMDB0010384	LysoPC(18:0/0:0)	C26H54NO7P	523.3637896	M+Na	+	3.906069134	UP
659.5009	HMDB0007161	DG(18:0/18:2(9Z,12Z)/0:0)	C39H72O5	620.5379754	M+K	+	0.234306324	DOWN
661.5164	HMDB0007159	DG(18:0/18:1(11Z)/0:0)	C39H74O5	622.5536255	M+K	+	0.149779163	DOWN
683.5008	HMDB0007171	DG(18:0/20:4(8Z,11Z,14Z,17Z)/0:0)	C41H72O5	644.5379754	M+K	+	0.062739574	DOWN
724.5251	HMDB0011342	PE(P-16:0/18:1(9Z))	C39H76NO7P	701.5359403	M+Na	+	0.267765191	DOWN
728.5588	HMDB0011440	PE(P-18:1(9Z)/18:1(11Z))	C41H78NO7P	727.5515904	M+H	+	0.069874918	DOWN

© 2024. Cheng Y et al. Published online at http://www.aginganddisease.org/EN/10.14336/AD.2024.1444

730.5731	HMDB0011439	PE(P-18:1(9Z)/18:0)	C41H80NO7P	729.5672404	M+H	+	0.185732368	DOWN
744.5896	HMDB0011210	PC(P-16:0/18:1(9Z))	C42H82NO7P	743.5828905	M+H	+	0.25800828	DOWN
760.5848	HMDB0007972	PC(16:0/18:1(9Z))	C42H82NO8P	759.5778051	M+H	+	0.469417724	DOWN
772.6204	HMDB0011307	PC(P-18:1(9Z)/18:0)	C44H86NO7P	771.6141906	M+H	+	0.139639495	DOWN
778.5721	HMDB0011447	PE(P-18:1(9Z)/20:1(11Z))	C43H82NO7P	755.5828905	M+Na	+	0.198629463	DOWN
780.588	HMDB0011446	PE(P-18:1(9Z)/20:0)	C43H84NO7P	757.5985406	M+Na	+	0.283429219	DOWN
786.5995	HMDB0008039	PC(18:0/18:2(9Z,12Z))	C44H84NO8P	785.5934552	M+H	+	0.189120205	DOWN
788.6159	HMDB0008037	PC(18:0/18:1(11Z))	C44H86NO8P	787.6091052	M+H	+	0.159597948	DOWN
832.582	HMDB0008048	PC(18:0/20:4(5Z,8Z,11Z,14Z))	C46H84NO8P	809.5934552	M+Na	+	0.281921983	DOWN
218.1024	HMDB0000210	Pantothenic acid	C9H17NO5	219.1106727	M-H	-	2.850312754	UP
244.1369	HMDB0059773	S-3-oxodecanoyl cysteamine	C12H23NO2S	245.1449497	M-H	-	0.105240479	DOWN
253.0924	HMDB0304157	3-beta-D-galactosyl-sn-glycerol	C9H18O8	254.1001676	M-H	-	4.550082896	UP
256.0588	HMDB0000803	beta-N-Acetylglucosamine	C8H15NO6	221.0899372	M+Cl	-	7.239254051	UP
277.2171	HMDB0001388	alpha-Linolenic acid	C18H30O2	278.2245802	M-H	-	7.996321737	UP
279.2323	HMDB0000673	Linoleic acid	C18H32O2	280.2402303	M-H	-	2.861279866	UP
281.2479	HMDB0000207	Oleic acid	C18H34O2	282.2558803	M-H	-	2.808812824	UP
301.2171	HMDB0001999	Eicosapentaenoic acid	C20H30O2	302.2245802	M-H	-	27.44676421	UP
305.248	HMDB0010378	5,8,11-Eicosatrienoic acid	C20H34O2	306.2558803	M-H	-	3.395828742	UP
307.2636	HMDB0005060	Eicosadienoic acid	C20H36O2	308.2715304	M-H	-	5.521049759	UP
313.2385	HMDB0004705	12,13-DHOME	C18H34O4	314.2457096	M-H	-	8.507025561	UP
329.2487	HMDB0001976	Docosapentaenoic acid (22n-6)	C22H34O2	330.2558803	M-H	-	8.835582446	UP
331.2639	HMDB0002226	Adrenic acid	C22H36O2	332.2715304	M-H	-	5.010999669	UP
335.2225	HMDB0002982	Prostaglandin B1	C20H32O4	336.2300595	M-H	-	3.207511635	UP
343.2278	HMDB0060049	4-HDoHE	C22H32O3	344.2351449	M-H	-	12.55532032	UP
357.2799	HMDB0258880	Tetracosapentenoic acid	C24H38O2	358.2871805	M-H	-	13.04235781	UP
365.246	HMDB0011564	MG(16:0/0:0/0:0)	C19H38O4	330.2770097	M+Cl	-	3.003446139	UP
365.3419	HMDB0002368	Nervonic acid	C24H46O2	366.3497807	M-H	-	2.847111139	UP
509.288	HMDB0240602	LysoPG(18:1(9Z)/0:0)	C24H47O9P	510.2957701	M-H	-	0.135912702	DOWN
600.5129	HMDB0004950	Cer(d18:1/18:0)	C36H71NO3	565.5433951	M+Cl	-	4.172901285	UP
619.2889	HMDB0061690	LysoPI(20:4(5Z,8Z,11Z,14Z)/0:0)	C29H49O12P	620.2961635	M-H	-	0.352235932	DOWN
673.4796	HMDB0007858	PA(16:0/18:1(11Z))	C37H71O8P	674.4886562	M-H	-	0.062204624	DOWN
701.5103	HMDB0114926	PA(18:1(9Z)/18:0)	C39H75O8P	702.5199564	M-H	-	0.040209989	DOWN
702.5421	HMDB0011157	PE(O-16:0/18:1(9Z))	C39H78NO7P	703.5515904	M-H	-	4.179544037	UP
723.4945	HMDB0114884	PA(18:0/20:4(5Z,8Z,11Z,14Z))	C41H73O8P	724.5043063	M-H	-	0.166362865	DOWN

© 2024. Cheng Y et al. Published online at http://www.aginganddisease.org/EN/10.14336/AD.2024.1444

742.539	HMDB0008994	PE(18:0/18:2(9Z,12Z))	C41H78NO8P	743.546505	M-H	-	0.25366098	DOWN
744.5538	HMDB0008992	PE(18:0/18:1(11Z))	C41H80NO8P	745.5621551	M-H	-	0.349351006	DOWN
750.544	HMDB0011449	PE(P-18:1(9Z)/20:3(5Z,8Z,11Z))	C43H78NO7P	751.5515904	M-H	-	0.384517579	DOWN
764.52	HMDB0009037	PE(18:1(11Z)/20:4(8Z,11Z,14Z,17Z))	C43H76NO8P	765.5308549	M-H	-	0.284882397	DOWN
786.5286	HMDB0012380	PS(18:0/18:2(9Z,12Z))	C42H78NO10P	787.5363342	M-H	-	0.114703984	DOWN
788.5435	HMDB0112289	PS(18:0/18:1(11Z))	C42H80NO10P	789.5519848	M-H	-	0.142185544	DOWN
809.5075	HMDB0244089	1,2-Dioleoyl-sn-glycero-3-phosphoglycerol	C42H79O10P	774.5410857	M+Cl	-	0.163363706	DOWN
810.5301	HMDB0012383	PS(18:0/20:4(5Z,8Z,11Z,14Z))	C44H78NO10P	811.5363342	M-H	-	0.217243954	DOWN
838.5588	HMDB0112382	PS(18:0/22:4(7Z,10Z,13Z,16Z))	C46H82NO10P	839.5676348	M-H	-	0.171491942	DOWN
842.5907	HMDB0112401	PS(18:1(11Z)/22:1(13Z))	C46H86NO10P	843.598935	M-H	-	0.282636329	DOWN
844.6062	HMDB0112713	PS(22:0/18:1(11Z))	C46H88NO10P	845.614585	M-H	-	0.201309046	DOWN
857.5174	HMDB0009789	PI(16:0/20:4(5Z,8Z,11Z,14Z))	C45H79O13P	858.5258296	M-H	-	0.340907941	DOWN
862.6073	HMDB0012316	3-O-Sulfogalactosylceramide (d18:1/22:0)	C46H89NO11S	863.6156334	M-H	-	0.181192429	DOWN
863.5628	HMDB0240667	PI(18:0/18:1(9Z))	C45H85O13P	864.5727793	M-H	-	0.365884142	DOWN
865.5015	HMDB0116605	PG(22:6(4Z,7Z,10Z,13Z,16Z,19Z)/22:6(4Z,7Z,10Z,13Z,16Z,19Z))	C50H75O10P	866.5097856	M-H	-	14.35142004	UP
885.5478	HMDB0009815	PI(18:0/20:4(5Z,8Z,11Z,14Z))	C47H83O13P	886.5571297	M-H	-	0.350468399	DOWN
888.6239	HMDB0012318	3-O-Sulfogalactosylceramide (d18:1/24:1(15Z))	C48H91NO11S	889.6312834	M-H	-	0.168624082	DOWN

Supplementary Table 4. 38 differential metabolites in thalamus in LPC-D7.

m/z	Compound id	Compound name	formula	Monoisotopic mass	adduct	Adduct type	FC	Changes
116.0709	HMDB0000162	Proline	C5H9NO2	115.0633	M+H	+	14.82166	UP
118.0866	HMDB0000043	Betaine	C5H12NO2	118.0868	M+H	+	190.1184	UP
126.0222	HMDB0000251	Taurine	C2H7NO3S	125.0147	M+H	+	1.686196	UP
130.0501	HMDB0000267	Pyroglutamic acid	C5H7NO3	129.0426	M+H	+	1.458833	UP
146.0927	HMDB0003464	4-Guanidinobutanoic acid	C5H11N3O2	145.0851	M+H	+	0.656115	DOWN
147.0764	HMDB0000641	Glutamine	C5H10N2O3	146.0691	M+H	+	1.640696	UP
156.0422	HMDB0000883	L-Valine	C5H11NO2	117.079	M+K	+	2.354536	UP
162.1125	HMDB0000062	L-Carnitine	C7H15NO3	161.1052	M+H	+	1.702371	UP
164.0084	HMDB0000224	O-Phosphoethanolamine	C2H8NO4P	141.0191	M+Na	+	1.691932	UP
189.0525	HMDB0000779	Phenyllactic acid	C9H10O3	166.063	M+Na	+	1.723174	UP
203.1392	HMDB0028690	Alanylisoleucine	C9H18N2O3	202.1317	M+H	+	3.417624	UP
204.1232	HMDB0000201	L-Acetylcarnitine	C9H17NO4	203.1158	M+H	+	8.167884	UP

© 2024. Cheng Y et al. Published online at http://www.aginganddisease.org/EN/10.14336/AD.2024.1444

206.0555	HMDB0001565	Phosphorylcholine	C5H15NO4P	184.0739	M+Na	+	2.761566	UP
229.1546	HMDB0304810	Pro-Ile	C11H20N2O3	228.1474	M+H	+	4.612767	UP
241.1294	HMDB0000745	Homocarnosine	C10H16N4O3	240.1222	M+H	+	0.633158	DOWN
259.0942	HMDB0011737	gamma-Glutamylglutamic acid	C10H16N2O7	276.0958	M+H-H2O	+	1.744126	UP
268.1039	HMDB0000050	Adenosine	C10H13N5O4	267.0968	M+H	+	0.206984	DOWN
293.1149	HMDB0029104	Tyrosyl-Glutamate	C14H18N2O6	310.1165	M+H-H2O	+	2.110282	UP
401.2662	HMDB0011579	MG(20:4(8Z,11Z,14Z,17Z)/0:0/0:0)	C23H38O4	378.277	M+Na	+	0.543367	DOWN
441.2402	HMDB0011587	MG(22:6(4Z,7Z,10Z,13Z,16Z,19Z)/0:0/0:0)	C25H38O4	402.277	M+K	+	0.507265	DOWN
740.498	HMDB0011342	PE(P-16:0/18:1(9Z))	C39H76NO7P	701.5359	M+K	+	0.435787	DOWN
766.5134	HMDB0011440	PE(P-18:1(9Z)/18:1(11Z))	C41H78NO7P	727.5516	M+K	+	0.529383	DOWN
768.5272	HMDB0011439	PE(P-18:1(9Z)/18:0)	C41H80NO7P	729.5672	M+K	+	0.519078	DOWN
824.5561	HMDB0008039	PC(18:0/18:2(9Z,12Z))	C44H84NO8P	785.5935	M+K	+	0.704865	DOWN
866.6467	HMDB0252577	Galactocerebrosides	C48H93NO9	827.685	M+K	+	0.537261	DOWN
199.0377	HMDB0002712	1,5-Anhydrosorbitol	C6H12O5	164.0685	M+Cl	-	2.349257	UP
229.0111	HMDB0001548	D-Ribose 5-phosphate	C5H11O8P	230.0192	M-H	-	0.563739	DOWN
251.1285	HMDB0036047	3alpha-Hydroxyoreadone	C14H20O4	252.1362	M-H	-	3.169305	UP
282.084	HMDB0000133	Guanosine	C10H13N5O5	283.0917	M-H	-	0.242728	DOWN
295.2275	HMDB0004667	13-HODE	C18H32O3	296.2351	M-H	-	1.482997	UP
303.083	HMDB0001067	N-Acetylaspartylglutamic acid	C11H16N2O8	304.0907	M-H	-	0.676489	DOWN
335.2225	HMDB0002982	Prostaglandin B1	C20H32O4	336.2301	M-H	-	1.542194	UP
346.0556	HMDB0000045	Adenosine monophosphate	C10H14N5O7P	347.0631	M-H	-	11.22961	UP
357.2805	HMDB0258880	Tetracosapentenoic acid	C24H38O2	358.2872	M-H	-	2.96932	UP
598.4969	HMDB0011774	Cer(d18:1/18:1(11Z))	C36H69NO3	563.5277	M+Cl	-	4.472097	UP
600.5131	HMDB0004950	Cer(d18:1/18:0)	C36H71NO3	565.5434	M+Cl	-	1.927571	UP
619.2886	HMDB0061690	LysoPI(20:4(5Z,8Z,11Z,14Z)/0:0)	C29H49O12P	620.2962	M-H	-	0.564885	DOWN
722.5097	HMDB0011444	PE(P-18:1(9Z)/18:3(9Z,12Z,15Z))	C41H74NO7P	723.5203	M-H	-	1.655822	UP

© 2024. Cheng Y et al. Published online at http://www.aginganddisease.org/EN/10.14336/AD.2024.1444
